# Nationwide surveillance, mitochondrial genetic diversity, and associated microorganisms of *Lipoptena fortisetosa* collected from cervids in the Republic of Korea

**DOI:** 10.1016/j.ijppaw.2026.101304

**Published:** 2026-10-15

**Authors:** Jong-Uk Jeong, Jeong-Min Kim, Hae-Eun Kang, SeEun Choe

**Affiliations:** Foreign Animal Disease Division, Animal and Plant Quarantine Agency, Gimcheon-si, Gyeongsangbuk-do, 39660, Republic of Korea

**Keywords:** *Lipoptena fortisetosa*, Deer ked, COX1, *Bartonella*, *Theileria*, Wildlife surveillance, One health

## Abstract

**Background:**

Deer keds are blood-feeding ectoparasites of cervids and can carry diverse microorganisms of veterinary or potential zoonotic relevance. Nationwide information on their distribution, mitochondrial diversity, and associated microorganisms has been lacking in the Republic of Korea (ROK). This study investigated deer keds collected from cervids across the ROK and characterized their species identity, COX1 diversity, and sequence-confirmed microorganisms.

**Methods:**

A total of 405 deer keds were opportunistically collected from 116 cervids made available through ongoing regional wildlife management activities across nine provinces of the Republic of Korea, including Korean water deer (*Hydropotes inermis*) on the mainland and Siberian roe deer (*Capreolus pygargus*) on Jeju Island. All 405 specimens were examined morphologically, and 186 were analyzed using cytochrome oxidase subunit 1 (COX1) sequencing for molecular confirmation and population-genetic analysis. Selected viral, bacterial, and protozoan microorganisms were screened using pooled qPCR/qRT-PCR or conventional PCR, followed by individual conventional PCR and Sanger sequencing for confirmation. Host-level clustering was evaluated using binomial generalized linear mixed models when sufficient confirmed detections were available.

**Results:**

All 405 deer keds were identified morphologically as *Lipoptena fortisetosa*, and all 186 COX1-sequenced specimens were molecularly consistent with this identification. The 617-bp COX1 dataset comprised 29 haplotypes (Hd = 0.681 ± 0.038; π = 0.01262 ± 0.00101). AMOVA showed significant mitochondrial differentiation between Jeju Island and mainland samples (ΦST = 0.615, P < 0.001), although host species and geography were completely confounded. Sequence-confirmed microorganisms included *Bartonella* spp. in 94/405 deer keds (23.21%), *Theileria* spp. in 40/405 (9.88%), *Coxiella*-like organisms in 4/405 (0.99%), and *Anaplasma* capra in 2/405 (0.49%). No sequence-confirmed *Babesia* spp., *Ehrlichia* spp., *Borrelia* spp., *Hepatozoon* spp., or SFTSV were obtained. *Bartonella* detection showed very strong host-level clustering and a marked Jeju-mainland contrast, whereas *Theileria* detection did not differ significantly between regions after accounting for host clustering.

**Conclusions:**

*Lipoptena fortisetosa* was widely associated with the cervids examined across the ROK and showed substantial mitochondrial genetic structure between the sampled Jeju and mainland groups. *Bartonella* spp. were the most frequently sequence-confirmed microorganisms, followed by *Theileria* spp. Molecular detection alone does not establish vector competence, and the geographic and host-species effects in the present sampling design cannot be separated. Standardized longitudinal sampling and host-vector-microorganism interaction studies are required to clarify transmission ecology and population structure.

## Highlights

• Deer keds from 116 cervids across nine Korean provinces were surveyed.
• All 405 deer keds were *L. fortisetosa*; COX1 confirmed 186 specimens.
• COX1 analysis found 29 haplotypes and Jeju-mainland genetic differentiation.
• Host species and geography were confounded in the Jeju-mainland comparison.
• *Bartonella* was most frequently detected, followed by *Theileria*.

## Graphical abstract

**Figure:**
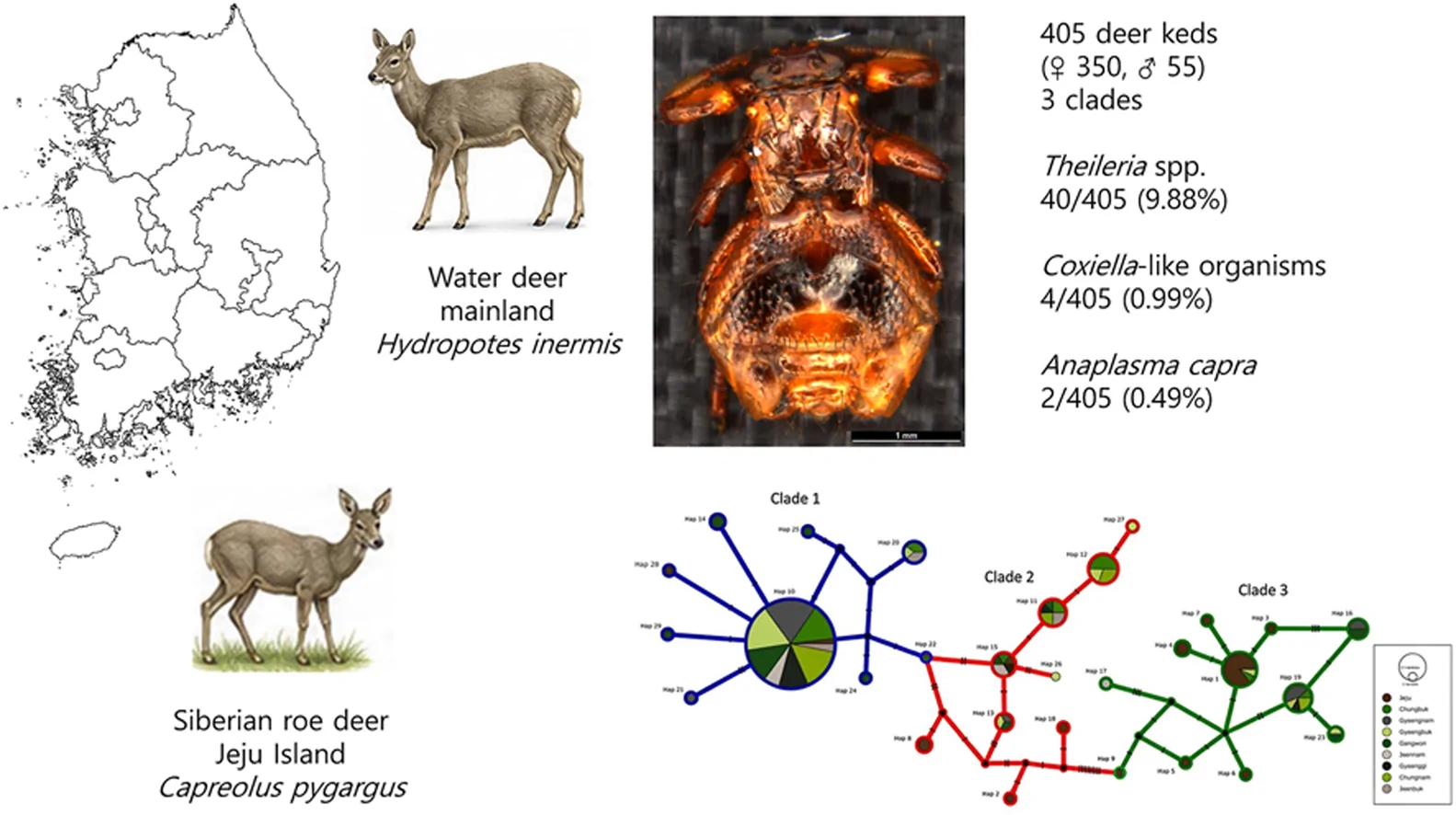

## 1 Background

Deer keds (Diptera: Hippoboscidae, *Lipoptena* spp.) are obligate blood-feeding ectoparasites of wild mammals, particularly cervids, and are widely distributed throughout Europe and Asia. Approximately 30 *Lipoptena* species have been described worldwide. Published records from the Republic of Korea (ROK) document *L. cervi* and *L. fortisetosa* (Yamauchi et al., 2009; Choi et al., 2013; Iwasa and Choi, 2013). Unlike actively host-seeking hematophagous insects such as mosquitoes, newly emerged deer keds typically remain on vegetation until a suitable host passes nearby, after which they fly only a short distance before attaching (Kortet et al., 2010; Dibo et al., 2023). Following attachment, they shed their wings and spend most of their adult lives on the host, feeding repeatedly on blood and reproducing through adenotrophic viviparity (Yamauchi et al., 2009; Dibo et al., 2023). Because deer keds parasitize free-ranging cervids that may interact with livestock and human environments, they are relevant surveillance targets at the wildlife-livestock-human interface.

Beyond causing irritation, dermatitis, and occasional anemia in heavily infested animals (Allan, 2001), deer keds have attracted increasing attention because DNA or RNA of numerous microorganisms have been detected in these insects. Previous studies have reported *Anaplasma* spp., *Bartonella* spp., *Borrelia* spp., *Coxiella*-like organisms, *Theileria* spp., *Trypanosoma* spp., *Rickettsia* spp., and severe fever with thrombocytopenia syndrome virus (SFTSV) in deer keds (Korhonen et al., 2015; Lee et al., 2016; Werszko et al., 2020; Gałęcki et al., 2021; Tiawsirisup et al., 2023; Casel et al., 2025). Although these findings indicate exposure to microorganisms circulating in wildlife systems, molecular detection alone does not demonstrate biological vector competence, which requires evidence of pathogen survival, replication, and transmission between hosts. Nevertheless, deer keds represent useful targets for surveillance of microorganisms associated with cervid systems.

In the ROK, the two principal cervid hosts are the water deer (*Hydropotes inermis*), which is widely distributed throughout the mainland, and the Siberian roe deer (*Capreolus pygargus*), which is abundant on Jeju Island. Increasing cervid populations and expanding contact between wildlife, livestock, and human environments may increase opportunities for humans and domestic animals to encounter deer keds and the pathogens detected in these ectoparasites. Consequently, understanding the current distribution of deer keds and the pathogen DNA detected in them has become increasingly important from both veterinary and public health perspectives.

Historically, *L. cervi* was reported from Korean water deer on the mainland (Iwasa and Choi, 2013). *L. fortisetosa* was subsequently recorded from Jeju Island, first as a single specimen of unknown host origin and later in large numbers from Siberian roe deer (Yamauchi et al., 2009; Choi et al., 2013). A later mainland study identified *L. fortisetosa* from a road-killed Korean water deer and detected *Coxiella*-like bacteria and *Theileria* DNA (Lee et al., 2016). More recently, SFTSV RNA was detected in *L. fortisetosa* collected from wild animals in central ROK (Casel et al., 2025). These studies established the presence of *L. fortisetosa* and several associated microorganisms in Korea but were geographically restricted, leaving its nationwide occurrence, mitochondrial diversity, and broader microorganism profile insufficiently characterized.

In this study, we conducted nationwide surveillance of deer keds collected from cervids across nine provinces of the ROK during 2025. We performed morphological identification and COX1-based molecular analysis, characterized mitochondrial genetic diversity and population structure, and screened for selected viral, bacterial, and protozoan microorganisms using sequence confirmation as the basis for final detection. The study was designed to provide updated baseline information on deer ked occurrence and genetic diversity while accounting for the clustered host-based sampling structure in the analysis of associated microorganisms.

## 2 Materials and methods

### 2.1 Sample collection

Deer keds were collected from cervids made available through ongoing regional wildlife management activities in the Republic of Korea. The host animals were not captured or euthanized specifically for this study. Sampling was conducted in collaboration with the Wildlife Management Association, and individual host animals within each participating province were examined when they became available during the study period.

Between June and November 2025, deer keds were collected from 116 cervids across nine provinces of the Republic of Korea: Gangwon-do (GW), Gyeonggi-do (GG), Gyeongsangnam-do (GN), Gyeongsangbuk-do (GB), Jeollanam-do (JN), Jeollabuk-do (JB), Jeju-do (JJ), Chungcheongnam-do (CN), and Chungcheongbuk-do (CB). On the mainland, deer keds were collected from Korean water deer (*Hydropotes inermis*), whereas those collected on Jeju Island were obtained from Siberian roe deer (*Capreolus pygargus*). A total of 405 deer keds were obtained, comprising 315 specimens from mainland regions and 90 specimens from Jeju Island.

Available host information, including sex and collection locality, was recorded at the time of sampling. Because exact geographic coordinates were not consistently available for harvested animals, collection locations were recorded at the available administrative level and are presented as approximate city- or county-level locations in Fig. 1. Deer keds were removed from the pelage of harvested cervids using sterile forceps, transported to the laboratory, and stored at −70°C until morphological identification and molecular analysis.

**Fig. 1:**
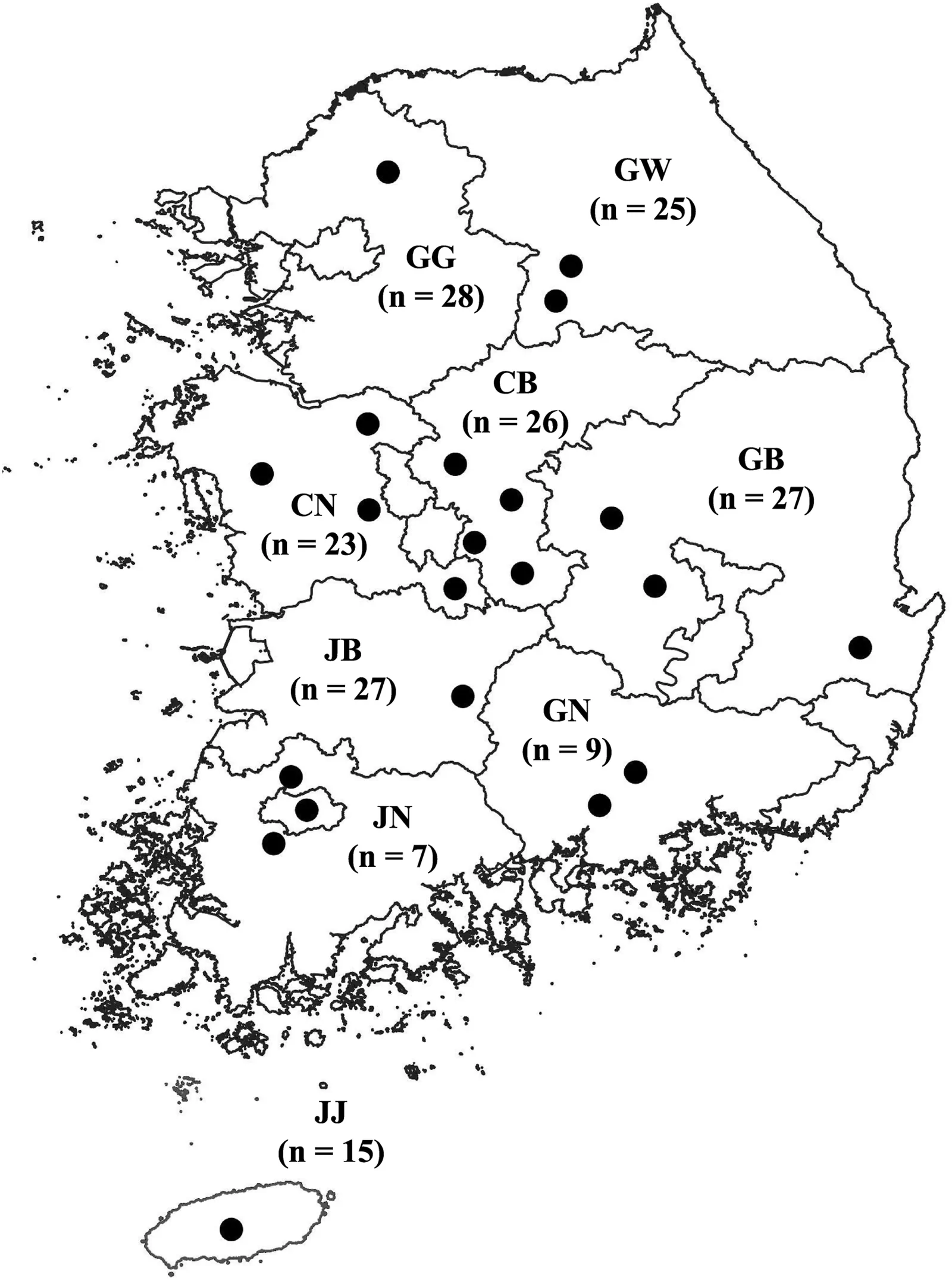
Geographic distribution of deer ked collection localities in the Republic of Korea in 2025. Black circles (●) indicate approximate city- or county-level sampling localities rather than exact collection coordinates. Numbers in parentheses indicate the number of cervid hosts from which deer keds were collected in each province. For Jeju-do, a single representative provincial-level point is shown because more detailed locality information was unavailable. Province abbreviations: GW, Gangwon-do; GG, Gyeonggi-do; GN, Gyeongsangnam-do; GB, Gyeongsangbuk-do; JN, Jeollanam-do; JB, Jeollabuk-do; JJ, Jeju-do; CN, Chungcheongnam-do; CB, Chungcheongbuk-do.

### 2.2 Morphological identification

All 405 deer keds were identified morphologically to species and sex under a stereo microscope (Olympus SZ61, Olympus, Japan) according to published taxonomic keys (Maa, 1967; Iwasa and Choi, 2013; Yatsuk et al., 2024). Specimens with uncertain or ambiguous morphological characteristics were additionally subjected to COX1 sequencing for molecular confirmation. Because these specimens were processed together with the population-genetic sequencing set and were not separately enumerated in the contemporaneous laboratory records, an exact retrospective count could not be reliably reconstructed.

### 2.3 DNA extraction and molecular identification

Individual deer keds were homogenized in α-MEM supplemented with Antibiotic-Antimycotic solution using a bead homogenizer, and total nucleic acids were extracted with the Maxwell RSC Viral Total Nucleic Acid Purification Kit (Promega, Madison, WI, USA) according to the manufacturer's instructions. For COX1 amplification, the Folmer primers LCO1490 and HCO2198 (Folmer et al., 1994) were used (Table 1), producing an approximately 710-bp amplicon. PCR products were commercially Sanger sequenced, bidirectional sequences were assembled and trimmed in ChromasPro 2.1.4, and consensus sequences were compared with GenBank reference sequences using BLAST for molecular confirmation.

**Table 1:** Primers used for molecular identification and conventional PCR confirmation of selected microorganisms in deer ked samples.

Purpose	Target gene	Primer		Primer sequence (5′→3′)	Expected size (bp)	Reference
Molecular identification	mtCOX1	LCO1490		GGT CAA CAA ATC ATA AAG ATA TTG G	710	Folmer et al. (1994)
		HCO2198		TAA ACT TCA GGG TGA CCA AAA AAT CA		
SFTSV	M	1st	m805f	ACT GGG CCG TGT TCT GAA TCA GA	600	Yun et al. (2014)
			M1409R	TAT CCA AGG AGG ATG ACA ATA AT		
		2nd	M811F	GAA GAG ACT TGC AAG ACC AGT GGT	450	
			M1240R	CCA CCA AAG CTC CTT TGA CGT GTA		
*Anaplasma* spp./*Ehrlichia* spp.	16S rRNA	AE1-F		AAG CTT AAC ACA TGC AAG TCG AA	1406	Oh et al. (2009)
		AE1-R		AGT CAC TGA CCC AAC CTT AAA TG		
Babesia spp./Theileria spp.	18S rRNA	1st	RLBF2	GAC ACA GGG AGG TAG TGA CAA G	500-1750	Schnittger et al. (2004)
		1st/2nd	RLBR2	CTA AGA ATT TCA CCT CTG ACA GT		
		2nd	RLB FIN	GAC AAG AAA TAA CAA TAC RGG GC		
*Borrelia* spp.	5S-23S rDNA	1st	bb23s3	CGA CCT TCT TCG CCT TAA AGC	412	Chu et al. (2008)
			bb23sa	TAA GCT GAC TAA TAC TAA TTA CCC		
		2nd	bb23s3nf	CTG CGA GTT CGC GGG AGA	226-266	
			bb23sanr	TCC TAG GCA TTC ACC ATA		
*Coxiella* spp.	16S rRNA	1st/2nd	Cox16SF1	CGT AGG AAT CTA CCT TRT AGW GG		Duron et al. (2014)
		1st	Cox16SR2	GCC TAC CCG CTT CTG GTA CAA TT	1321-1414	
		2nd	Cox16SR1	ACT YYC CAA CAG CTA GTT CTC A	719-813	
*Bartonella* spp.	rpoB	1400F		CGC ATT GGC TTA CTT CGT ATG	850	Renesto et al. (2001)
		2300R		GTA GAC TGA TTA GAA CGC TG		
*Hepatozoon* spp.	18S rRNA	HepF		ATA CAT GWG CAM AWT CTC AAC	666	Otranto et al. (2011)
		HepR		TTA TWA TTC CAT GCT GCA G		
*Bartonella* spp.	gltA	Screening	gltA BhCS.781p	GGGGACCAGCTCATGGTGG	379	Hammerbauerová et al. (2025)
			gltA BhCS.1137n	AATGCAAAAAGAACAGTAAACA		
		Sequencing	gltA 443F-D	GCYATGTCTGCATTYTATCA	790	
			gltA 1210R	GATCYTCAATCATTTCTTTCCA		

### 2.4 Population genetic analysis

For COX1 genotyping, up to 30 deer keds were selected from each province. All specimens were analyzed in provinces where 30 or fewer deer keds were collected. In provinces with more than 30 specimens, selection was designed to maximize representation of individual cervid hosts; when multiple deer keds were available from the same host, one specimen was randomly selected whenever possible. COX1 sequences already generated for specimens requiring molecular confirmation because of uncertain morphology were retained and counted toward the provincial maximum. This procedure yielded 186 COX1 sequences. The population-genetic alignment comprised 617 nucleotide positions with no gaps or missing data. Haplotype number, haplotype diversity (Hd), nucleotide diversity (π), segregating sites (S), mean pairwise nucleotide differences (k), Tajima's D, Fu's Fs, Fu and Li's D* and F*, and Ramos-Onsins and Rozas' R2 were calculated in DnaSP v6 (Rozas et al., 2017). A TCS haplotype network was generated in PopART (Clement et al., 2002; Leigh and Bryant, 2015). Population differentiation was assessed by analysis of molecular variance (AMOVA) among provinces and between the Jeju and mainland groups. Because all Jeju specimens originated from *C. pygargus* and all mainland specimens from *H. inermis*, the latter grouping represents a combined geographic/host-species contrast and was not interpreted as an independent effect of either factor.

### 2.5 Molecular screening and confirmation of selected microorganisms

Customized qPCR/qRT-PCR assay panels manufactured by KOREAGENETECH (Chuncheon, ROK) were used for initial screening. The assays targeted the S and M genomic segments of SFTSV (CR109), the 18S rRNA genes of *Babesia* spp. and *Theileria* spp. (BR241), and the 16S rRNA genes of *Anaplasma* spp., *Ehrlichia* spp., and *Borrelia* spp. (CR308). Primer and probe sequences were proprietary and were not disclosed by the manufacturer. Reactions were run on a Bio-Rad CFX Opus 96 instrument using 15 μL of assay premix and 5 μL of extracted nucleic acid (20 μL total). Positive and no-template controls were included in each run. According to the laboratory interpretation criteria, target Ct values < 40 were considered screening-reactive. Each pool was tested once because assay material was limited; reactive pools were not routinely re-tested by the same real-time PCR assay but were immediately de-pooled, and the constituent specimens were examined individually by conventional PCR.

To conserve assay material, BR241 and CR109 screening used two deer keds per pool and comprised 203 pools each. CR308 screening included 375 deer keds in 91 valid pools: 88 pools contained four specimens each, and the final three pools contained 8, 8, and 7 specimens, respectively. We examined all constituent specimens from screening-reactive pools individually by conventional PCR. We performed conventional PCR and nested PCR assays using the primer sets and amplification conditions described in the corresponding published protocols cited in Table 1. Every specimen yielding an expected-size conventional PCR band was subjected to Sanger sequencing, and only sequence-confirmed results were considered final detections.

### 2.6 Direct conventional PCR and Bartonella re-evaluation

*Coxiella*-like organisms and *Hepatozoon* spp. were investigated by conventional PCR using the primer sets listed in Table 1. *Coxiella* detection targeted the 16S rRNA gene and was analyzed independently from *C. burnetii*, which was not tested by qPCR in this study. The original *Bartonella* rpoB assay yielded no expected-size amplicons. Because primer compatibility may influence the detection of genetically diverse wildlife-associated *Bartonella*, all 405 deer keds were subsequently re-evaluated using gltA-based assays following Hammerbauerová et al. (2025). A 379-bp gltA assay was used for prescreening; specimens with expected-size products were then tested with a longer, approximately 790-bp gltA assay for characterization. All expected-size long-fragment products were Sanger sequenced. Chromatograms showing overlapping peaks at polymorphic sites were retained as Bartonella detections but excluded from single-genotype phylogenetic assignment.

### 2.7 Phylogenetic and statistical analyses

Maximum-likelihood phylogenetic analyses were conducted in MEGA12 (Kumar et al., 2024) using the Tamura-Nei nucleotide substitution model, uniform rates among sites, complete deletion of sites containing gaps or missing data, nearest-neighbor interchange, and 1000 bootstrap replicates. The final common alignments used for phylogenetic analysis were 577 bp for COX1, 1029 bp for *Anaplasma* 16S rRNA, 680 bp for *Coxiella*-like organism 16S rRNA, and 293 bp for *Theileria* 18S rRNA. *Bartonella* gltA sequences with clean chromatograms were collapsed into sequence types for phylogenetic visualization. Sequence-confirmed detection proportions were calculated for individual deer keds. We also calculated the proportion of examined cervid hosts from which at least one sequence-confirmed positive deer ked was collected, with Wilson 95% confidence intervals. For microorganisms with sufficient numbers of detections, binomial generalized linear mixed models were fitted in R using glmmTMB, with host identity as a random intercept and region (Jeju versus mainland) as a fixed effect. Because host species and region were completely confounded, the regional term was interpreted only as a combined host/geographic contrast. Rare detections were summarized descriptively rather than modeled inferentially.

## 3 Results

### 3.1 Deer ked identification

A total of 405 deer keds were obtained from 116 cervid hosts across nine provinces during 2025 (Table 2). The collection comprised 315 deer keds from 101 water deer on the mainland and 90 deer keds from 15 Siberian roe deer on Jeju Island. Among all deer keds, 350 (86.4%) were female and 55 (13.6%) were male.

**Table 2:** Number of *Lipoptena fortisetosa* collected from cervid hosts by province in this study.

Province	Host	Number of host individuals	Number of deer keds		
			Male	Female	Total
Gangwon-do	*Hydropotes inermis*	12	6	51	57
Gyeonggi-do		4	2	15	17
Gyeongsangnam-do		21	13	47	60
Gyeongsangbuk-do		12	8	35	43
Jeollanam-do		9	2	7	9
Jeollabuk-do		4	3	6	9
Chungcheongnam-do		18	4	51	55
Chungcheongbuk-do		21	13	52	65
Jeju-do	*Capreolus pygargus*	15	4	86	90
Total		116	55	350	405

All 405 specimens were morphologically identified as *Lipoptena fortisetosa*. COX1 sequencing was performed for 186 specimens included in molecular confirmation and population-genetic analysis, and all sequenced specimens were consistent with *L. fortisetosa*. No other *Lipoptena* species were identified in the present collection.

### 3.2 Genetic diversity analysis

The 186-sequence COX1 dataset comprised 617 bp, 34 segregating sites, 35 mutations, six singleton variable sites, 28 parsimony-informative sites, and 29 haplotypes. Overall haplotype diversity was 0.681 ± 0.038, nucleotide diversity was 0.01262 ± 0.00101, and the mean number of nucleotide differences was 7.786. Jeju specimens (n = 27) showed Hd = 0.803 ± 0.077 and π = 0.01399 ± 0.00234, whereas mainland specimens (n = 159) showed Hd = 0.587 ± 0.046 and π = 0.00900 ± 0.00113. Hap 10 was the most frequent haplotype and occurred across all nine provinces.

The haplotype network showed an uneven distribution of mitochondrial haplotypes between Jeju and mainland samples, and the COX1 maximum-likelihood analysis resolved three descriptive mitochondrial clades (Clades 1–3). These clade designations were used to summarize haplotype relationships and were not interpreted as independent geographic or host-associated lineages (Fig. 2, Fig. 3). Province-based AMOVA indicated significant differentiation (ΦST = 0.303, P < 0.001), with 30.32% of molecular variance among provinces and 69.68% within provinces. The Jeju-versus-mainland grouping showed stronger differentiation (ΦST = 0.615, P < 0.001), with 61.48% of variance between groups and 38.52% within groups. Because host species and geographic origin were completely confounded, this result describes differentiation between the sampled groups rather than an independent geographic or host-species effect.

**Fig. 2:**
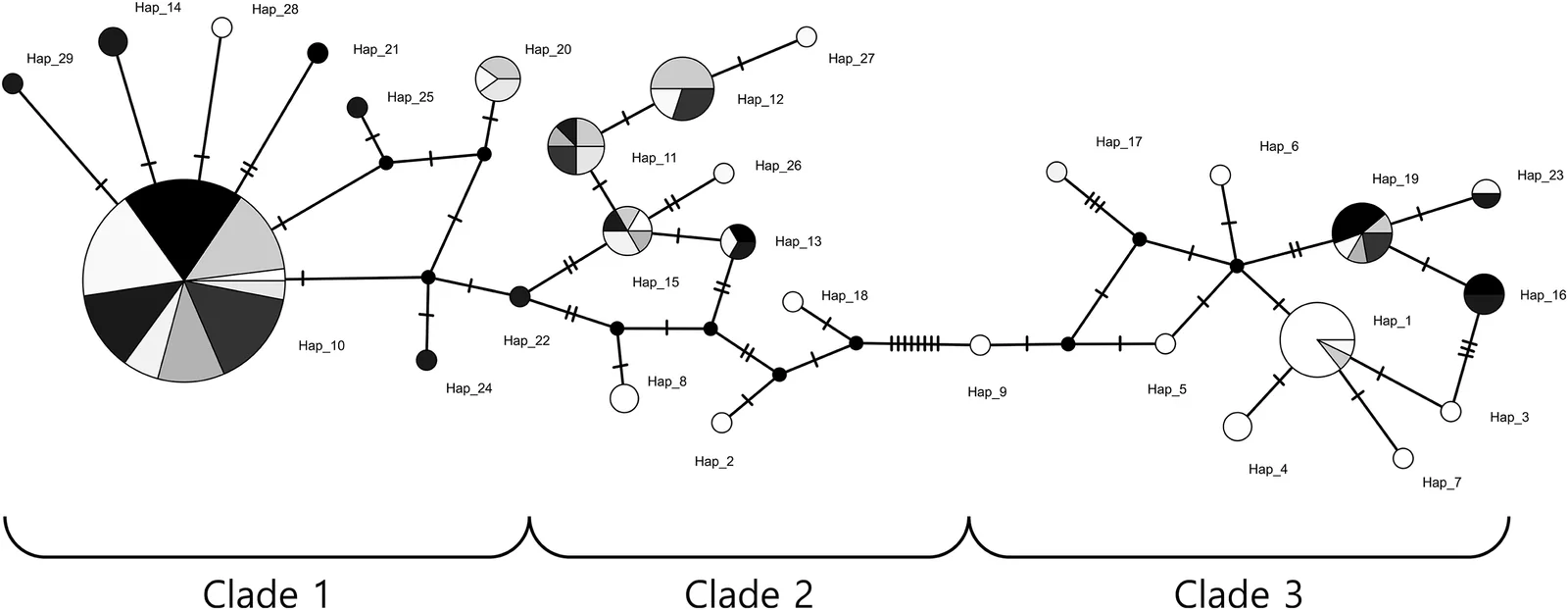
TCS haplotype network of *Lipoptena fortisetosa* based on 186 mitochondrial COX1 sequences. Circle size is proportional to haplotype frequency, sectors indicate provincial origin, hatch marks indicate mutational steps, and small black circles represent hypothetical intermediate haplotypes. The network illustrates mitochondrial haplotype composition across the sampled Korean populations without implying an independent host-species effect.

**Fig. 3:**
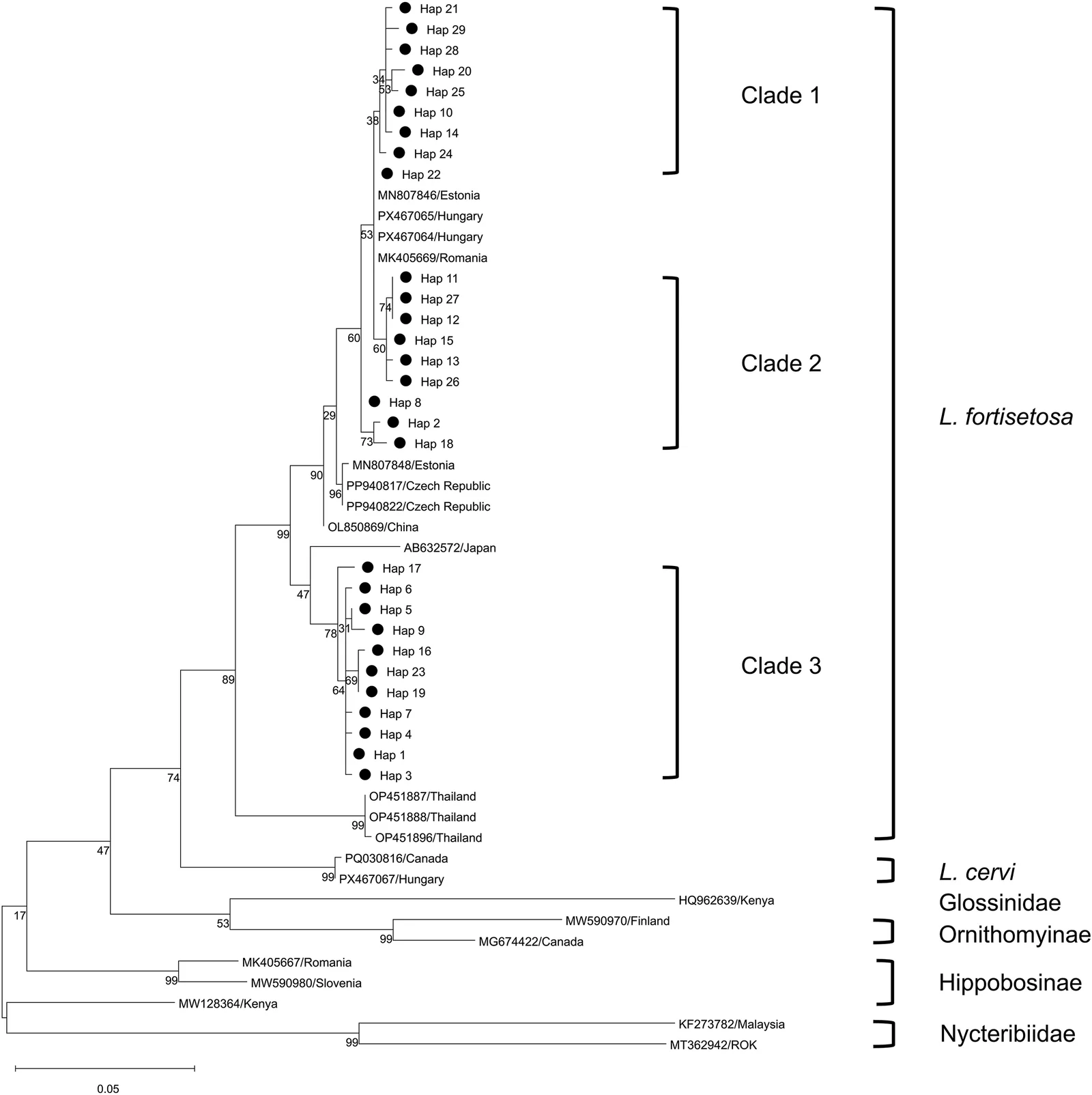
Maximum-likelihood phylogenetic tree of *Lipoptena fortisetosa* based on a 577-bp common COX1 alignment including reference sequences. Study haplotypes are marked with black circles (●). Numbers at nodes indicate bootstrap support based on 1000 replicates. The tree was used to visualize intraspecific mitochondrial relationships and species-level placement of the study haplotypes.

Neutrality statistics did not indicate a significant departure from neutral expectations or clear evidence of recent demographic expansion. For the full dataset, Tajima's D was 0.841, Fu's Fs was −1.184, and R2 was 0.1136 (P = 0.882). Jeju and mainland datasets likewise showed non-significant neutrality statistics. The 186 COX1 sequences were deposited in GenBank under accession numbers PZ852184-PZ852369.

### 3.3 Sequence-confirmed detection of selected microorganisms

Sequence-confirmed microorganisms were detected in deer keds at the following rates: *Bartonella* spp., 94/405 (23.21%, 95% CI 19.36-27.56%); *Theileria* spp., 40/405 (9.88%, 95% CI 7.34-13.17%); *Coxiella*-like organisms, 4/405 (0.99%, 95% CI 0.38-2.51%); and *Anaplasma capra*, 2/405 (0.49%, 95% CI 0.14-1.78%). At the host level, at least one sequence-confirmed positive deer ked was collected from 37/116 hosts for *Bartonella* (31.90%, 95% CI 24.11-40.84%), 28/116 for *Theileria* (24.14%, 95% CI 17.26-32.67%), 4/116 for *Coxiella*-like organisms (3.45%, 95% CI 1.35-8.53%), and 2/116 for *A. capra* (1.72%, 95% CI 0.47-6.07%). No sequence-confirmed *Babesia* spp., *Ehrlichia* spp., *Borrelia* spp., *Hepatozoon* spp., or SFTSV were obtained (Table 3).

**Table 3:** Sequence-confirmed detection of selected microorganisms in 405 deer keds collected from 116 cervid hosts.

Microorganism	Screening result	Expected-size cPCR amplicons n/N	Sequence-confirmed n/N (%, 95% CI)	Hosts with sequence-positive deer keds n/N (%, 95% CI)
*Bartonella* spp.	cPCR; 186/405	95/186	94/405 (23.21%, 19.36-27.56)	37/116 (31.90%, 24.11-40.84)
*Theileria* spp.	qPCR; 93/203 (pool) (186 samples)	169/186	40/405 (9.88%, 7.34-13.17)	28/116 (24.14%, 17.26-32.67)
*Anaplasma* spp.	qPCR; 13/91 (pool) (56 samples)	3/56	2/405 (0.49%, 0.14-1.78)	2/116 (1.72%, 0.47-6.07)
*Coxiella*-like organisms	cPCR; 5/405	-	4/405 (0.99%, 0.38-2.51)	4/116 (3.45%, 1.35-8.53)
SFTSV	qRT-PCR; 8/203 (pool) (16 samples)	0/16	0/405 (0%, 0-0.94)	0/116 (0%, 0-3.21)
*Ehrlichia* spp.	qPCR; 4/91 (pool) (16 samples);	0/16	0/405 (0%, 0-0.94)	0/116 (0%, 0-3.21)
*Borrelia* spp.	qPCR; 12/91 (pool) (48 samples)	1/48	0/405 (0%, 0-0.94)	0/116 (0%, 0-3.21)
*Babesia* spp.	qPCR; 0/203 (pool)	-	0/405 (0%, 0-0.94)	0/116 (0%, 0-3.21)
*Hepatozoon* spp.	cPCR; 0/405	-	0/405 (0%, 0-0.94)	0/116 (0%, 0-3.21)

Final detection was based on sequence confirmation. In the “Hosts with sequence-positive deer keds” column, n/N represents the number of cervid hosts yielding at least one sequence-confirmed positive deer ked divided by the total number of cervid hosts examined. Confidence intervals are Wilson 95% intervals. Screening-reactive results without conventional PCR and/or sequence confirmation were not classified as confirmed detections.

For *Anaplasma* spp., 13 of 91 qPCR-screened pools were reactive, comprising 56 individual deer keds. After de-pooling, three specimens (DK-0008, DK-0039, and DK-0046) yielded expected-size 16S rRNA amplicons, and all three were sequenced. DK-0008 and DK-0039 were identified as *A. capra*, whereas DK-0046 yielded insufficient-quality sequence for reliable identification. The two *A. capra* sequences were 1029 bp and were deposited in GenBank under accession numbers PZ854640-PZ854641 (Fig. 4).

**Fig. 4:**
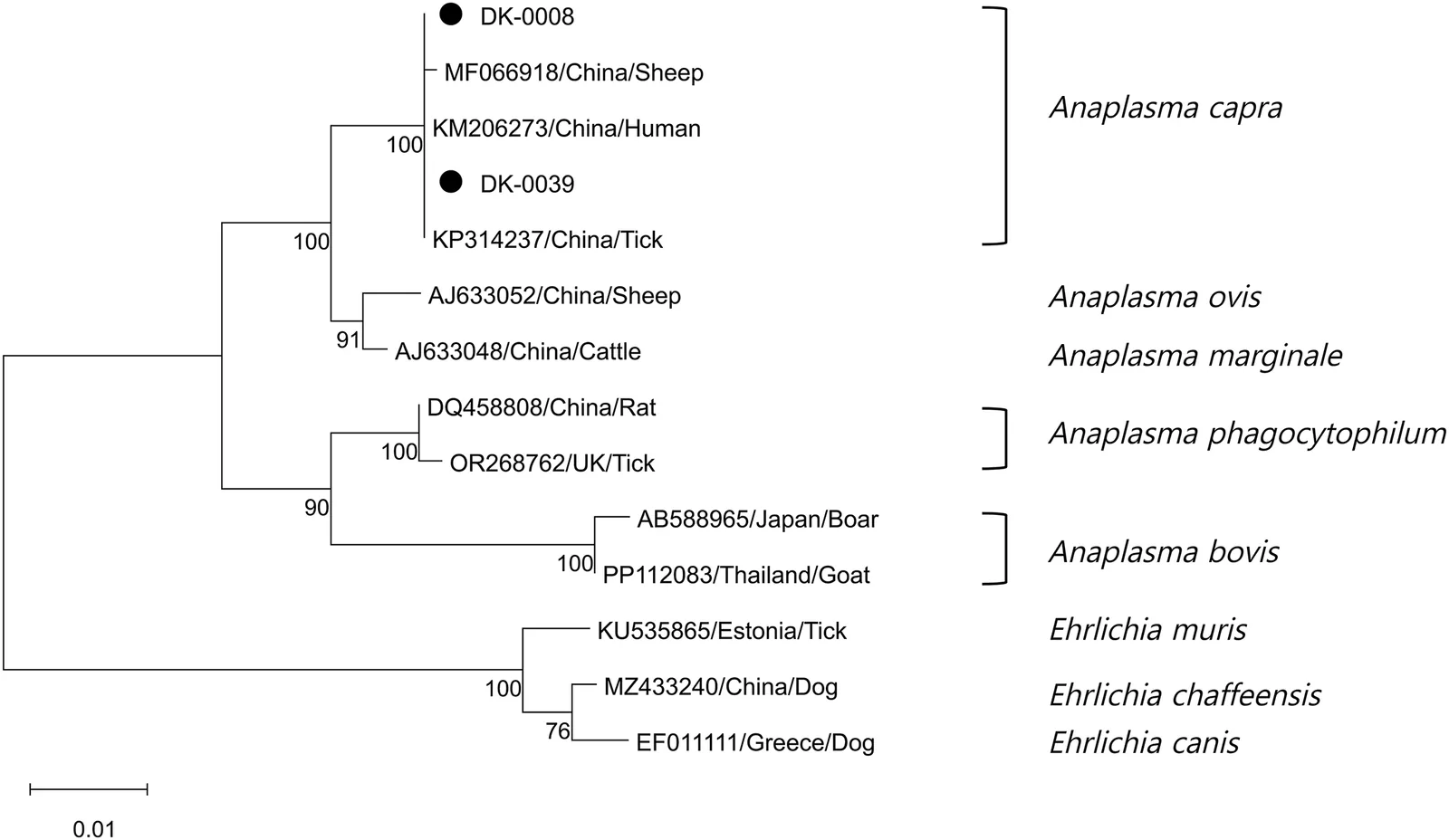
Maximum-likelihood phylogenetic tree of *Anaplasma* spp. based on a 1029-bp 16S rRNA alignment. DK-0008 and DK-0039, marked with black circles (●), represent *A. capra* sequences identified in this study. Numbers at nodes indicate bootstrap support based on 1000 replicates.

Five specimens yielded expected-size *Coxiella* 16S rRNA amplicons, and all five were sequenced. Four 680-bp sequences were confirmed as *Coxiella*-like organisms, whereas one yielded a non-target sequence. The four *Coxiella*-like sequences clustered within an arthropod-associated *Coxiella*-like lineage rather than with *C. burnetii* and were deposited under accession numbers PZ862439 and PZ889904-PZ889906 (Fig. 5).

**Fig. 5:**
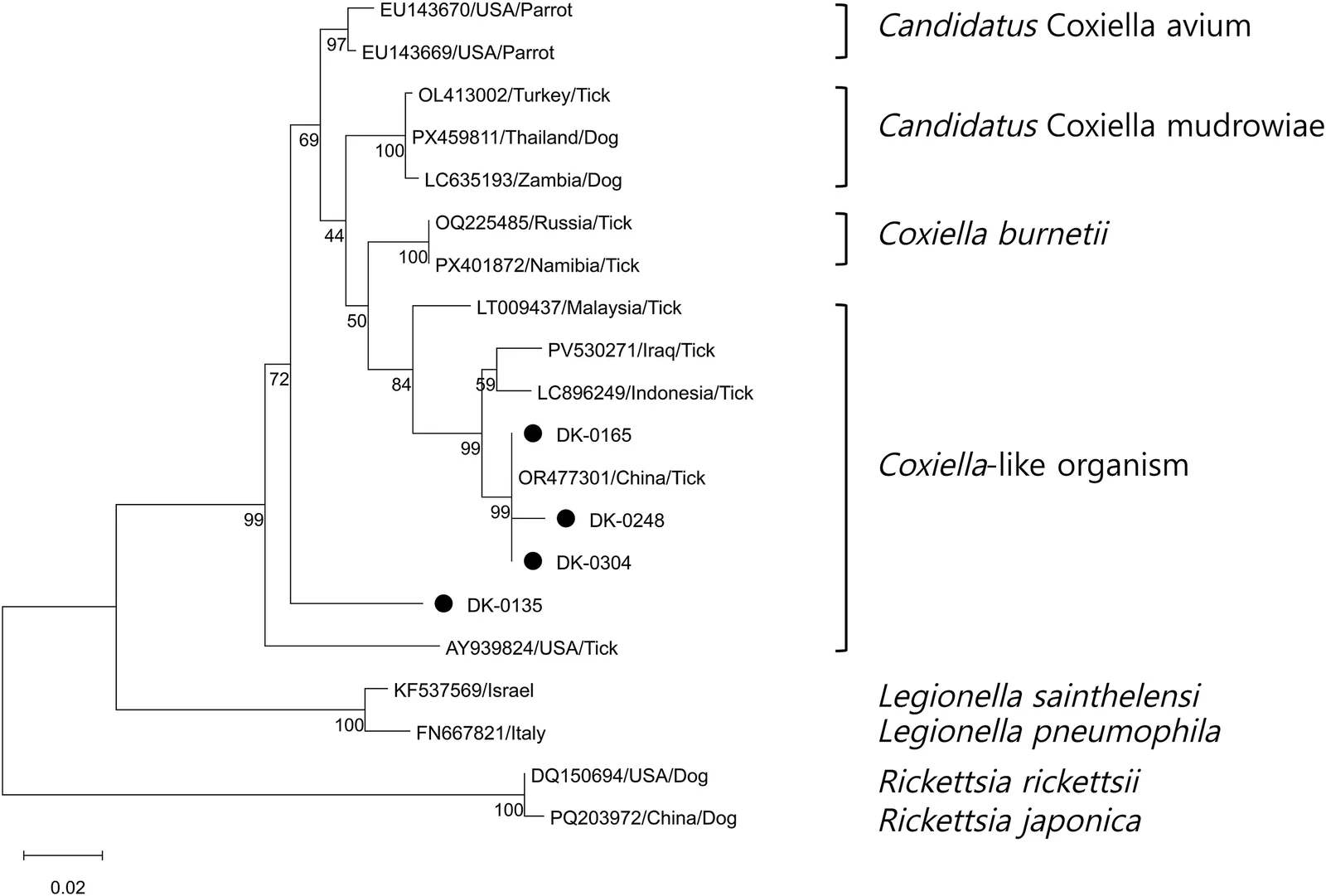
Maximum-likelihood phylogenetic tree of *Coxiella*-like organisms based on a 680-bp 16S rRNA alignment. DK-0135, DK-0165, DK-0248, and DK-0304 are marked with black circles (●). The four study sequences clustered within an arthropod-associated *Coxiella*-like lineage distinct from *C. burnetii*. Numbers at nodes indicate bootstrap support based on 1000 replicates.

For *Theileria* spp., 93 of 203 qPCR-screened pools were reactive, corresponding to 186 individual deer keds. Of these, 169/186 specimens yielded expected-size 18S rRNA amplicons by conventional PCR, and all 169 amplicons were subjected to Sanger sequencing. Of the 169 expected-size 18S rRNA amplicons subjected to Sanger sequencing, 40 yielded interpretable *Theileria* sequences and were considered sequence-confirmed detections. The remaining amplicons did not yield sequence data of sufficient quality for reliable taxonomic assignment and were therefore considered unconfirmed rather than qPCR false-positive results. Of the 40 sequence-confirmed detections, 35 sequences met the quality requirements for phylogenetic analysis and were included in the final 293-bp alignment.

Thirty-five *Theileria* 18S rRNA sequences of sufficient length and quality were retained for the final 293-bp phylogenetic analysis and were represented by seven sequence types. The topology showed broad affinity with *T. luwenshuni*-, *T. cervi*-, and *T. capreoli*-related lineages (Fig. 6). Because the analyzed 18S rRNA fragment was short and highly conserved, the tree was used primarily for broad phylogenetic placement rather than definitive species-level discrimination. The 35 sequences were deposited in GenBank under accession numbers PZ852376-PZ852410.

**Fig. 6:**
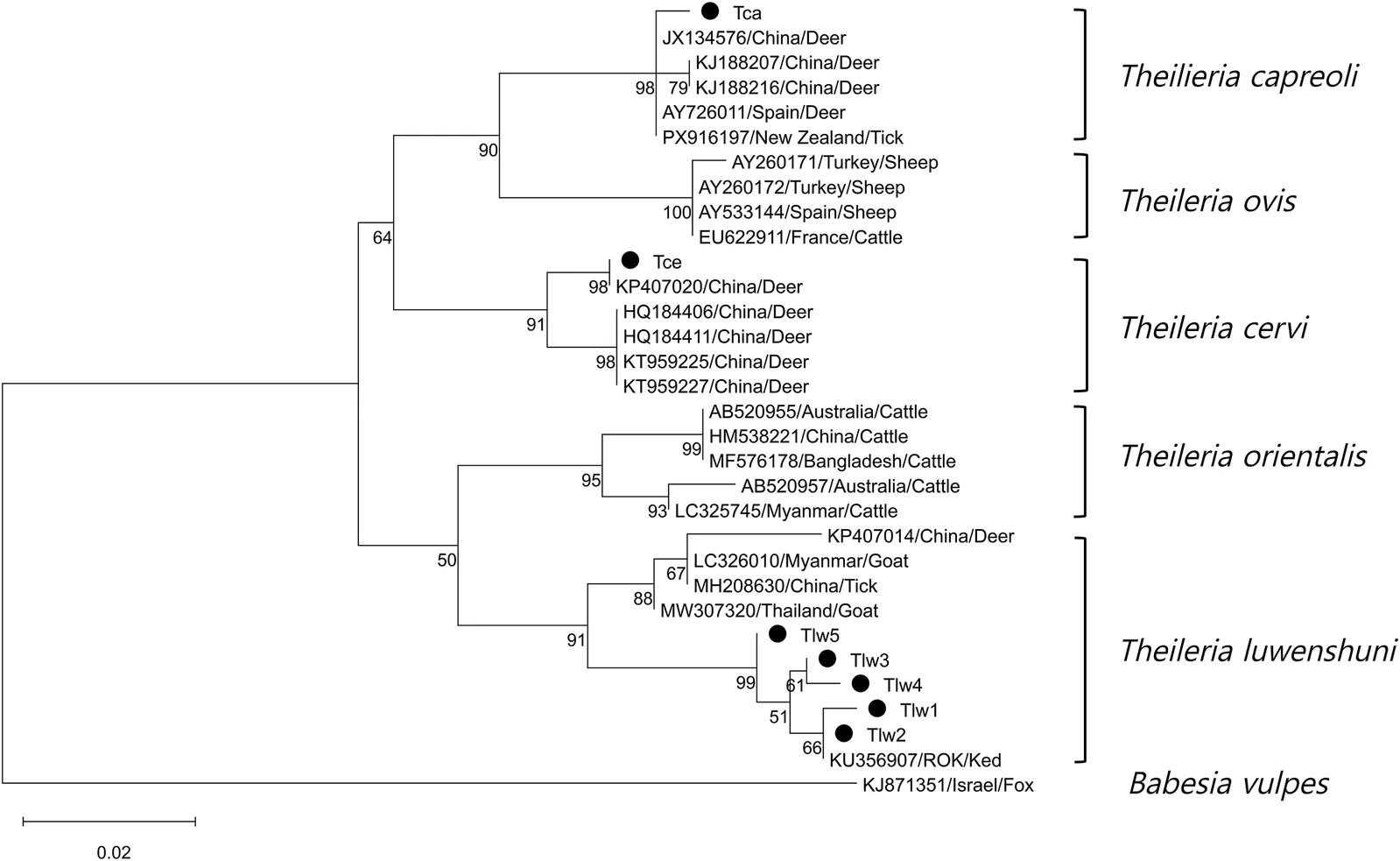
Maximum-likelihood phylogenetic tree of *Theileria* spp. based on a 293-bp 18S rRNA alignment. Thirty-five study sequences were represented by seven sequence types and are marked with black circles (●). Numbers at nodes indicate bootstrap support based on 1000 replicates. Because the analyzed fragment is short and highly conserved, the topology is intended for broad lineage placement rather than definitive species-level resolution.

For SFTSV, eight of 203 qRT-PCR-screened pools were reactive, comprising 16 deer keds; five pools were reactive for the M segment and three for the S segment. Contemporaneous laboratory records showed Ct values ranging from approximately 31.9 to 38.94. After de-pooling, all 16 specimens were negative by follow-up conventional PCR, and no SFTSV detection was sequence-confirmed.

Four of 91 qPCR-screened pools were reactive for *Ehrlichia* spp., corresponding to 16 specimens, all of which were negative by individual conventional PCR. Twelve of 91 qPCR-screened pools were reactive for *Borrelia* spp., corresponding to 48 specimens; one specimen yielded an expected-size conventional PCR amplicon, but sequencing was unsuccessful. No *Babesia*-reactive pools were detected among the 203 qPCR-screened pools.

Re-evaluation of *Bartonella* using gltA-based assays substantially changed the original result. The 379-bp gltA prescreening assay yielded expected-size products from 186/405 deer keds (45.9%). Of these, 95/186 yielded the expected approximately 790-bp gltA amplicon and all 95 were sequenced. Interpretable *Bartonella* sequences were obtained from 94 specimens (23.21%). Ninety-two chromatograms showed clean single-sequence profiles and were included in phylogenetic analysis; 50 clustered with *B. schoenbuchensis* reference sequences and 42 formed a distinct deer-ked-associated *Bartonella* lineage that was conservatively designated *Bartonella* spp. Two additional chromatograms showed overlapping A/G peaks at polymorphic positions differentiating *B. schoenbuchensis* sequence types, suggesting possible mixed *Bartonella* genotypes within individual deer keds. The 94 *Bartonella* gltA sequences (748 bp) were deposited in GenBank under accession numbers PZ986991-PZ987084 (Fig. 7).

**Fig. 7:**
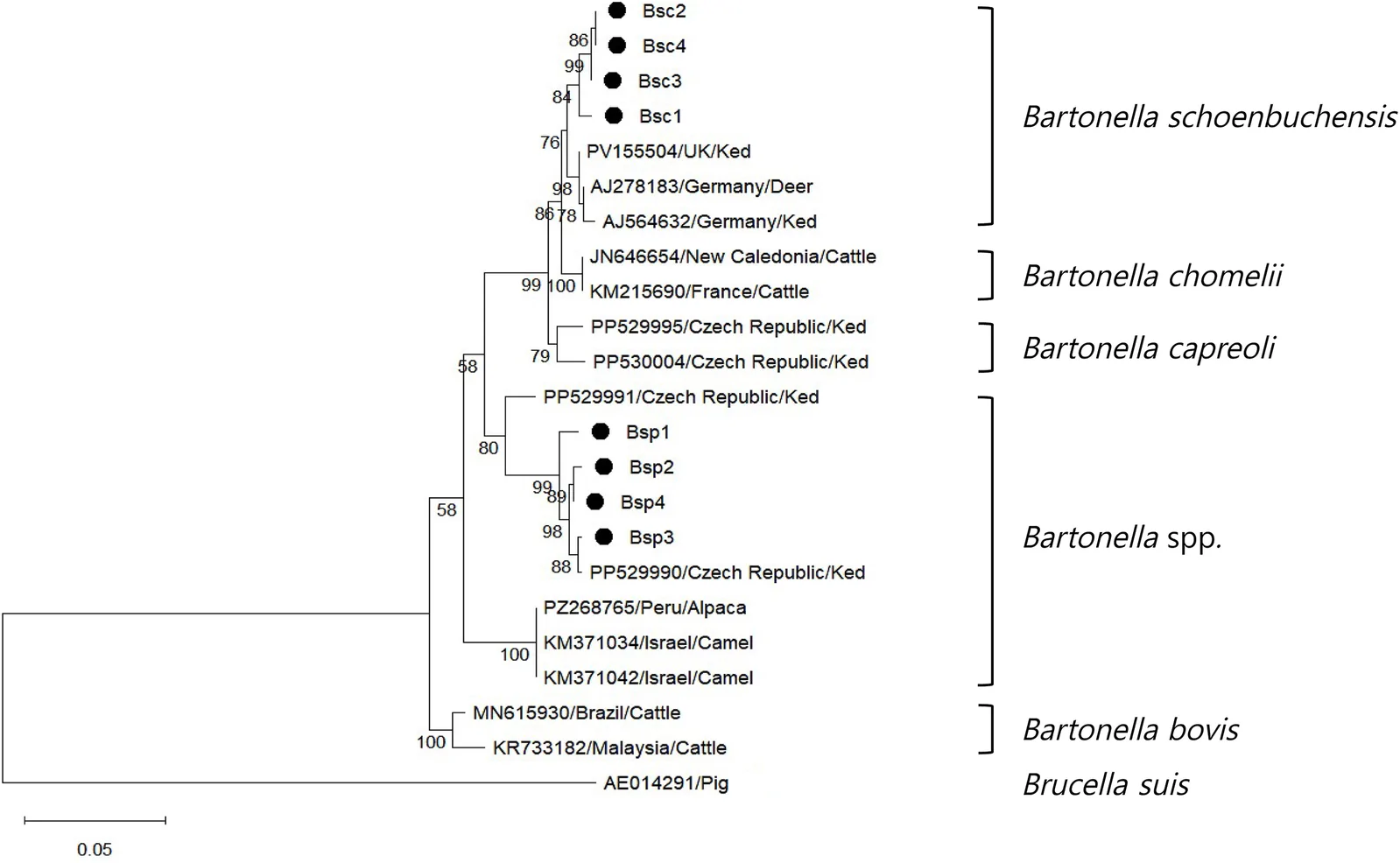
Maximum-likelihood phylogenetic tree of *Bartonella* based on the longer gltA fragment. Study sequence types are marked with black circles (●). Bsc1-Bsc4 clustered with *B*. *schoenbuchensis* reference sequences, whereas Bsp1-Bsp4 formed a distinct deer-ked-associated *Bartonella* lineage and were conservatively designated *Bartonella* spp. Two chromatograms showing mixed A/G peaks at polymorphic sites were excluded from single-genotype phylogenetic assignment. Numbers at nodes indicate bootstrap support values.

The sequence-confirmed *Bartonella* detections were strongly concentrated in the Jeju collection: 63/90 deer keds from Jeju were positive compared with 31/315 on the mainland. At the host level, 14/15 Jeju hosts and 23/101 mainland hosts had at least one positive deer ked. A binomial mixed-effects model showed very strong host-level clustering (latent-scale ICC = 0.98), and inclusion of region significantly improved model fit (likelihood-ratio χ^2^ = 16.19, P < 0.001; AIC 253.36 versus 267.55). The regional coefficient showed quasi-separation and generated an unstable, extremely large odds-ratio estimate; therefore, the odds ratio was not interpreted quantitatively. Because region and host species were completely confounded, this contrast cannot be attributed independently to geography or host species.

For *Theileria*, host-level clustering was also substantial (latent-scale ICC = 0.46). After accounting for host identity as a random intercept, detection did not differ significantly between Jeju and the mainland (OR = 1.28, 95% CI 0.31-5.23, P = 0.731). Inclusion of region did not improve model fit compared with the intercept-only model (likelihood-ratio χ^2^ = 0.12, P = 0.730; AIC 254.69 versus 252.80).

## 4 Discussion

The present study provides nationwide baseline information on *L. fortisetosa* collected from cervids in the ROK and integrates mitochondrial population genetics with sequence-confirmed microorganism surveillance. All deer keds examined morphologically were *L. fortisetosa*, and the 186 COX1-sequenced specimens were molecularly consistent with this identification. The study also revealed substantial mitochondrial differentiation between the sampled Jeju and mainland groups and identified *Bartonella* spp., *Theileria* spp., *A. capra*, and *Coxiella*-like organisms in subsets of deer keds.

Historically, *L. cervi* was reported from Korean water deer on the mainland, whereas *L. fortisetosa* was subsequently documented on Jeju Island and later from Korean water deer on the mainland (Yamauchi et al., 2009; Choi et al., 2013; Iwasa and Choi, 2013; Lee et al., 2016). The exclusive identification of *L. fortisetosa* in the present collection supports its widespread association with Korean cervids. However, the absence of *L. cervi* should not be interpreted as evidence of its disappearance from the ROK. Sampling was host-based and depended on animals available through ongoing wildlife management activities; host-seeking deer keds were not sampled, and the survey did not encompass all potential cervid hosts, seasons, or local ecological conditions. Species occurring at low abundance or showing different host associations or seasonal activity may therefore have been missed.

The COX1 dataset showed moderate overall mitochondrial diversity (Hd = 0.681; π = 0.01262), with higher diversity among Jeju specimens than among mainland specimens. The haplotype network, phylogenetic analysis, and AMOVA all indicated non-random mitochondrial structure between the sampled groups. In particular, the Jeju-versus-mainland AMOVA attributed 61.48% of molecular variance to differences between groups (ΦST = 0.615, P < 0.001). These findings indicate restricted mitochondrial genetic connectivity between the sampled groups but do not identify the mechanism responsible for that differentiation.

The biological basis of the observed COX1 differentiation cannot be resolved from the present sampling design because all deer keds from Jeju Island were collected from *C. pygargus*, whereas all mainland specimens were collected from *H. inermis*. Geographic origin and host species were therefore completely confounded. Geographic isolation of Jeju Island, restricted gene flow, founder effects, demographic history, and host-associated structuring are non-mutually exclusive possibilities, but the present data provide no basis for inferring host preference or host adaptation. None of the neutrality statistics showed significant evidence of recent demographic expansion or departure from neutral expectations. Sampling the same cervid species across geographic regions, or multiple sympatric cervid species within the same region, would be necessary to distinguish geographic from host-associated genetic structure.

*Bartonella* spp. were the most frequently sequence-confirmed microorganisms in this study. The contrast between the original negative rpoB-based assay and the subsequent gltA-based detection of Bartonella in 94 deer keds highlights the potential influence of primer suitability and assay design on the detection of genetically diverse wildlife-associated Bartonella. Hammerbauerová et al. (2025) also used gltA-based assays for the detection and characterization of wildlife-associated Bartonella. In the present study, *B. schoenbuchensis*-associated sequence types were strongly concentrated in Jeju samples, whereas the unclassified *Bartonella* lineage was more broadly distributed. Two samples also showed A/G mixed chromatogram peaks at sequence-type-specific SNP positions, consistent with possible mixed *Bartonella* genotypes. Direct Sanger sequencing cannot resolve the exact genotype composition of such mixtures, and this observation should therefore be interpreted as suggestive rather than definitive evidence of mixed infection.

*Bartonella* detection also showed pronounced clustering among deer keds collected from the same host and a marked Jeju-mainland contrast. However, the extreme imbalance between Jeju and mainland host-level detection produced quasi-separation in the mixed model, so the fitted odds ratio was not interpreted quantitatively. More importantly, the complete confounding of host species and geography precludes attribution of the contrast to either factor independently. Although frequent *Bartonella* detection indicates a close association between these bacteria and deer keds, molecular detection alone does not establish vector competence or transmission capability.

*Theileria* was sequence-confirmed in 40 deer keds. After accounting for clustering of multiple deer keds within individual cervid hosts, no significant Jeju-mainland difference was detected. The 293-bp 18S rRNA fragment provided broad phylogenetic placement with *T. luwenshuni*-, *T. cervi*-, and *T. capreoli*-related lineages, but its short length and high conservation limit species-level resolution. Accordingly, the *Theileria* topology should be interpreted as broad lineage placement rather than definitive species identification for closely related taxa. Longer 18S sequences and additional loci would improve taxonomic resolution.

*Anaplasma capra* was confirmed in two deer keds. *A. capra* is an emerging tick-borne zoonotic bacterium reported from domestic ruminants, ticks, wildlife, and humans (Liu et al., 2012; Li et al., 2015; Seo et al., 2018). Detection in deer keds extends the range of arthropod samples in which *A. capra* DNA has been observed in the ROK, but it does not establish biological transmission by deer keds.

Four deer keds contained *Coxiella*-like organism sequences that clustered with arthropod-associated *Coxiella* lineages rather than with *C. burnetii*. Such *Coxiella*-like organisms are commonly interpreted as putative endosymbionts in hematophagous arthropods rather than as confirmed pathogens (Duron et al., 2014). Their repeated detection is therefore of ecological and symbiotic interest, but their pathogenic significance should not be inferred from sequence detection alone.

No sequence-confirmed *Babesia* spp., *Ehrlichia* spp., *Borrelia* spp., *Hepatozoon* spp., or SFTSV were obtained. These findings should not be interpreted as evidence of absence because detection depends on host infection status, geographic and temporal exposure, template concentration, and assay performance. SFTSV RNA has previously been detected in Korean *L. fortisetosa* (Casel et al., 2025); in the present study, eight pools were qRT-PCR-reactive but none of the 16-constituent deer keds yielded a conventional PCR amplicon. *Ehrlichia* and *Borrelia* likewise produced screening-reactive pools without sequence confirmation. These results are therefore best described as unconfirmed screening reactions rather than established infections.

Several limitations should be considered when interpreting these findings. Deer keds were collected from cervids made available through ongoing wildlife management activities; therefore, the number of hosts available from each province and their within-province collection locations could not be predetermined by the investigators. This resulted in uneven regional sampling, with some provinces represented by relatively small numbers of hosts and deer keds. In addition, host species and geographic region were completely confounded because all Siberian roe deer were sampled on Jeju Island, whereas all Korean water deer were sampled on the mainland. Consequently, host-species and geographic effects could not be distinguished independently. The sampling design was also not suitable for estimating infestation intensity or seasonal abundance because field collectors removed visible deer keds directly from available hosts and the total number of parasites present on each animal could not be quantified reliably.

Initial screening was performed on pooled samples, and each pool was tested once because commercial assay material was limited. Reactive pools were de-pooled and followed immediately by individual conventional PCR, and every expected-size conventional PCR product was sequenced. Original SFTSV Ct values were available from contemporaneous laboratory records, whereas archived raw files for some other assays were later re-exported under altered baseline/threshold settings and did not fully reproduce the contemporaneous screening calls. Consequently, retrospectively recalculated Ct values were not substituted for the original screening records. Final microorganism detection was based on sequence confirmation rather than screening reactivity alone.

Finally, PCR-based detection identifies nucleic acid and does not demonstrate microorganism viability, replication, or biological transmission. The results should therefore not be interpreted as evidence that *L. fortisetosa* is a competent vector for the detected microorganisms.

## 5 Conclusion

This nationwide survey showed that *L. fortisetosa* was widely associated with the cervids examined across the ROK and revealed substantial mitochondrial genetic differentiation between the sampled Jeju and mainland groups. *Bartonella* spp. were the most frequently sequence-confirmed microorganisms, followed by *Theileria* spp., while *A. capra* and *Coxiella*-like organisms were detected less frequently. The geographic and host-species components of the observed differences could not be separated, and molecular detection does not establish vector competence. The results provide a baseline for standardized longitudinal surveillance and future studies of deer ked population connectivity and host-vector-microorganism interactions.

## CRediT authorship contribution statement

**Jong-Uk Jeong:** Writing – original draft, Methodology, Investigation, Formal analysis, Data curation, Conceptualization. **Jeong-Min Kim:** Investigation, Formal analysis, Data curation. **Hae-Eun Kang:** Funding acquisition, Conceptualization. **SeEun Choe:** Writing – review & editing, Validation, Supervision, Formal analysis.

## Ethics approval and consent to participate

The cervids from which deer keds were collected were legally harvested as part of ongoing regional wildlife management activities and were not captured, handled, or euthanized specifically for this study. Deer keds were opportunistically collected from the harvested cervids, and no live-animal procedures were performed by the investigators as part of this study.

## Consent for publication

Not applicable.

## Availability of data and materials

The datasets used and/or analyzed during the current study are available from the corresponding author upon reasonable request.

## Funding

This work was supported by the 10.13039/501100008771Animal and Plant Quarantine Agency of the Republic of Korea (Grant No. M-1543085-2025-26-02).

## Declaration of competing interest

The authors declare the following financial interests/personal relationships which may be considered as potential competing interests: SeEun Choe reports financial support was provided by Animal and Plant Quarantine Agency. If there are other authors, they declare that they have no known competing financial interests or personal relationships that could have appeared to influence the work reported in this paper.

## Abbreviations

AMOVA: analysis of molecular variance; CI: confidence interval; CLO: *Coxiella*-like organism; COX1: cytochrome oxidase subunit 1; GLMM: generalized linear mixed model; ICC: intraclass correlation coefficient; ML: maximum likelihood; qPCR: quantitative polymerase chain reaction; qRT-PCR: quantitative reverse transcription polymerase chain reaction; ROK: Republic of Korea; SFTSV: severe fever with thrombocytopenia syndrome virus.
