# Supplementary material for: Nationwide surveillance, mitochondrial genetic diversity, and associated microorganisms of Lipoptena fortisetosa collected from cervids in the Republic of Korea
Source: Int J Parasitol Parasites Wildl. 2026 Oct 15;31:101304. doi: 10.1016/j.ijppaw.2026.101304 (PMC13692108; doi:10.1016/j.ijppaw.2026.101304)
Supplement: Supplementary file 1 [file mmc1.docx]

**Supplementary Table S1.** Distribution of *Lipoptena fortisetosa* haplotypes by province.

| Haplotype | n | Clade | Gangwon-do | Gyeonggi-do | Gyeongsangnam-do | Gyeongsangbuk-do | Jeollanam-do | Jeollabuk-do | Jeju-do | Chungcheongnam-do | Chungcheongbuk-do |
| --- | --- | --- | --- | --- | --- | --- | --- | --- | --- | --- | --- |
| Hap 1 | 14 | 3 | 0 | 0 | 0 | 1 | 0 | 0 | 12 | 0 | 1 |
| Hap 2 | 1 | 2 | 0 | 0 | 0 | 0 | 0 | 0 | 1 | 0 | 0 |
| Hap 3 | 1 | 3 | 0 | 0 | 0 | 0 | 0 | 0 | 1 | 0 | 0 |
| Hap 4 | 2 | 3 | 0 | 0 | 0 | 0 | 0 | 0 | 2 | 0 | 0 |
| Hap 5 | 1 | 3 | 0 | 0 | 0 | 0 | 0 | 0 | 1 | 0 | 0 |
| Hap 6 | 1 | 3 | 0 | 0 | 0 | 0 | 0 | 0 | 1 | 0 | 0 |
| Hap 7 | 1 | 3 | 0 | 0 | 0 | 0 | 0 | 0 | 1 | 0 | 0 |
| Hap 8 | 2 | 2 | 0 | 0 | 0 | 0 | 0 | 0 | 2 | 0 | 0 |
| Hap 9 | 1 | 3 | 0 | 0 | 0 | 0 | 0 | 0 | 1 | 0 | 0 |
| Hap 10 | 103 | 1 | 13 | 11 | 20 | 18 | 6 | 3 | 2 | 16 | 14 |
| Hap 11 | 8 | 2 | 1 | 1 | 0 | 0 | 0 | 2 | 0 | 2 | 2 |
| Hap 12 | 10 | 2 | 0 | 0 | 0 | 2 | 0 | 0 | 0 | 3 | 5 |
| Hap 13 | 3 | 2 | 1 | 0 | 1 | 1 | 0 | 0 | 0 | 0 | 0 |
| Hap 14 | 2 | 1 | 2 | 0 | 0 | 0 | 0 | 0 | 0 | 0 | 0 |
| Hap 15 | 6 | 2 | 1 | 1 | 0 | 0 | 2 | 0 | 1 | 0 | 1 |
| Hap 16 | 4 | 3 | 2 | 0 | 2 | 0 | 0 | 0 | 0 | 0 | 0 |
| Hap 17 | 1 | 3 | 0 | 0 | 0 | 0 | 1 | 0 | 0 | 0 | 0 |
| Hap 18 | 1 | 2 | 0 | 0 | 0 | 0 | 0 | 0 | 1 | 0 | 0 |
| Hap 19 | 9 | 3 | 0 | 1 | 4 | 1 | 0 | 0 | 0 | 2 | 1 |
| Hap 20 | 5 | 1 | 0 | 0 | 0 | 1 | 0 | 2 | 0 | 0 | 2 |
| Hap 21 | 1 | 1 | 0 | 0 | 1 | 0 | 0 | 0 | 0 | 0 | 0 |
| Hap 22 | 1 | 1 | 1 | 0 | 0 | 0 | 0 | 0 | 0 | 0 | 0 |
| Hap 23 | 2 | 3 | 1 | 0 | 0 | 1 | 0 | 0 | 0 | 0 | 0 |
| Hap 24 | 1 | 1 | 1 | 0 | 0 | 0 | 0 | 0 | 0 | 0 | 0 |
| Hap 25 | 1 | 1 | 1 | 0 | 0 | 0 | 0 | 0 | 0 | 0 | 0 |
| Hap 26 | 1 | 2 | 0 | 0 | 0 | 1 | 0 | 0 | 0 | 0 | 0 |
| Hap 27 | 1 | 2 | 0 | 0 | 0 | 1 | 0 | 0 | 0 | 0 | 0 |
| Hap 28 | 1 | 1 | 0 | 0 | 0 | 0 | 0 | 0 | 1 | 0 | 0 |
| Hap 29 | 1 | 1 | 1 | 0 | 0 | 0 | 0 | 0 | 0 | 0 | 0 |
| Total | 186 |  | 25 | 14 | 28 | 27 | 9 | 7 | 27 | 23 | 26 |

**Supplementary Table S2.** AMOVA results for province-based and Jeju-versus-mainland COX1 groupings.

| Grouping / source of variation | df | SSD | MSD | Variance component | Variation (%) | ΦST | P-value |  |
| --- | --- | --- | --- | --- | --- | --- | --- | --- |
| A. Province-based AMOVA (9 provinces) | | | | | | | | |
| Among provinces | 8 | 3681.086 | 460.13578 | 20.367 | 30.32 | 0.303 | <0.001 |  |
| Within provinces | 177 | 8283.726 | 46.80071 | 46.801 | 69.68 |  |  |  |
| Total | 185 | 11964.812 | 64.67466 |  | 100.00 |  |  |  |
| B. Jeju-versus-mainland grouping | | | | | | | | |
| Between Jeju and mainland groups | 1 | 3454.092 | 3454.09205 | 73.825 | 61.48 | 0.615 | <0.001 |  |
| Within groups | 184 | 8510.720 | 46.25391 | 46.254 | 38.52 |  |  |  |
| Total | 185 | 11964.812 | 64.67466 |  | 100.00 |  |  |  |

AMOVA was based on 186 COX1 sequences (617 bp). P-values were obtained by permutation tests. The Jeju-versus-mainland grouping is completely confounded with host species (Jeju: *Capreolus pygargus*; mainland: *Hydropotes inermis*) and should therefore be interpreted as a combined geographic/host-species contrast.

**Supplementary Table S3.** Individual specimen identifiers, target genes, sequence lengths, and GenBank accession numbers for sequences generated in this study.

**A. COX1, *Anaplasma*, *Theileria*, and *Coxiella*-like organism sequences**

| Specimen ID | Sequence / organism | Target gene | Sequence length (bp) | GenBank accession no. |
| --- | --- | --- | --- | --- |
| DK-0001 | *Lipoptena fortisetosa* | COX1 | 617 | PZ852184 |
| DK-0002 | *Lipoptena fortisetosa* | COX1 | 617 | PZ852185 |
| DK-0005 | *Lipoptena fortisetosa* | COX1 | 617 | PZ852186 |
| DK-0006 | *Lipoptena fortisetosa* | COX1 | 617 | PZ852187 |
| DK-0007 | *Lipoptena fortisetosa* | COX1 | 617 | PZ852188 |
| DK-0008 | *Lipoptena fortisetosa* | COX1 | 617 | PZ852189 |
| DK-0009 | *Lipoptena fortisetosa* | COX1 | 617 | PZ852190 |
| DK-0010 | *Lipoptena fortisetosa* | COX1 | 617 | PZ852191 |
| DK-0011 | *Lipoptena fortisetosa* | COX1 | 617 | PZ852192 |
| DK-0012 | *Lipoptena fortisetosa* | COX1 | 617 | PZ852193 |
| DK-0013 | *Lipoptena fortisetosa* | COX1 | 617 | PZ852194 |
| DK-0015 | *Lipoptena fortisetosa* | COX1 | 617 | PZ852195 |
| DK-0016 | *Lipoptena fortisetosa* | COX1 | 617 | PZ852196 |
| DK-0017 | *Lipoptena fortisetosa* | COX1 | 617 | PZ852197 |
| DK-0018 | *Lipoptena fortisetosa* | COX1 | 617 | PZ852198 |
| DK-0019 | *Lipoptena fortisetosa* | COX1 | 617 | PZ852199 |
| DK-0020 | *Lipoptena fortisetosa* | COX1 | 617 | PZ852200 |
| DK-0021 | *Lipoptena fortisetosa* | COX1 | 617 | PZ852201 |
| DK-0022 | *Lipoptena fortisetosa* | COX1 | 617 | PZ852202 |
| DK-0023 | *Lipoptena fortisetosa* | COX1 | 617 | PZ852203 |
| DK-0024 | *Lipoptena fortisetosa* | COX1 | 617 | PZ852204 |
| DK-0025 | *Lipoptena fortisetosa* | COX1 | 617 | PZ852205 |
| DK-0026 | *Lipoptena fortisetosa* | COX1 | 617 | PZ852206 |
| DK-0027 | *Lipoptena fortisetosa* | COX1 | 617 | PZ852207 |
| DK-0028 | *Lipoptena fortisetosa* | COX1 | 617 | PZ852208 |
| DK-0029 | *Lipoptena fortisetosa* | COX1 | 617 | PZ852209 |
| DK-0030 | *Lipoptena fortisetosa* | COX1 | 617 | PZ852210 |
| DK-0031 | *Lipoptena fortisetosa* | COX1 | 617 | PZ852211 |
| DK-0032 | *Lipoptena fortisetosa* | COX1 | 617 | PZ852212 |
| DK-0033 | *Lipoptena fortisetosa* | COX1 | 617 | PZ852213 |
| DK-0034 | *Lipoptena fortisetosa* | COX1 | 617 | PZ852214 |
| DK-0035 | *Lipoptena fortisetosa* | COX1 | 617 | PZ852215 |
| DK-0036 | *Lipoptena fortisetosa* | COX1 | 617 | PZ852216 |
| DK-0037 | *Lipoptena fortisetosa* | COX1 | 617 | PZ852217 |
| DK-0038 | *Lipoptena fortisetosa* | COX1 | 617 | PZ852218 |
| DK-0039 | *Lipoptena fortisetosa* | COX1 | 617 | PZ852219 |
| DK-0040 | *Lipoptena fortisetosa* | COX1 | 617 | PZ852220 |
| DK-0041 | *Lipoptena fortisetosa* | COX1 | 617 | PZ852221 |
| DK-0042 | *Lipoptena fortisetosa* | COX1 | 617 | PZ852222 |
| DK-0043 | *Lipoptena fortisetosa* | COX1 | 617 | PZ852223 |
| DK-0044 | *Lipoptena fortisetosa* | COX1 | 617 | PZ852224 |
| DK-0045 | *Lipoptena fortisetosa* | COX1 | 617 | PZ852225 |
| DK-0046 | *Lipoptena fortisetosa* | COX1 | 617 | PZ852226 |
| DK-0048 | *Lipoptena fortisetosa* | COX1 | 617 | PZ852227 |
| DK-0049 | *Lipoptena fortisetosa* | COX1 | 617 | PZ852228 |
| DK-0050 | *Lipoptena fortisetosa* | COX1 | 617 | PZ852229 |
| DK-0051 | *Lipoptena fortisetosa* | COX1 | 617 | PZ852230 |
| DK-0052 | *Lipoptena fortisetosa* | COX1 | 617 | PZ852231 |
| DK-0053 | *Lipoptena fortisetosa* | COX1 | 617 | PZ852232 |
| DK-0054 | *Lipoptena fortisetosa* | COX1 | 617 | PZ852233 |
| DK-0055 | *Lipoptena fortisetosa* | COX1 | 617 | PZ852234 |
| DK-0056 | *Lipoptena fortisetosa* | COX1 | 617 | PZ852235 |
| DK-0057 | *Lipoptena fortisetosa* | COX1 | 617 | PZ852236 |
| DK-0058 | *Lipoptena fortisetosa* | COX1 | 617 | PZ852237 |
| DK-0059 | *Lipoptena fortisetosa* | COX1 | 617 | PZ852238 |
| DK-0062 | *Lipoptena fortisetosa* | COX1 | 617 | PZ852239 |
| DK-0063 | *Lipoptena fortisetosa* | COX1 | 617 | PZ852240 |
| DK-0064 | *Lipoptena fortisetosa* | COX1 | 617 | PZ852241 |
| DK-0067 | *Lipoptena fortisetosa* | COX1 | 617 | PZ852242 |
| DK-0069 | *Lipoptena fortisetosa* | COX1 | 617 | PZ852243 |
| DK-0070 | *Lipoptena fortisetosa* | COX1 | 617 | PZ852244 |
| DK-0071 | *Lipoptena fortisetosa* | COX1 | 617 | PZ852245 |
| DK-0072 | *Lipoptena fortisetosa* | COX1 | 617 | PZ852246 |
| DK-0073 | *Lipoptena fortisetosa* | COX1 | 617 | PZ852247 |
| DK-0074 | *Lipoptena fortisetosa* | COX1 | 617 | PZ852248 |
| DK-0075 | *Lipoptena fortisetosa* | COX1 | 617 | PZ852249 |
| DK-0076 | *Lipoptena fortisetosa* | COX1 | 617 | PZ852250 |
| DK-0077 | *Lipoptena fortisetosa* | COX1 | 617 | PZ852251 |
| DK-0078 | *Lipoptena fortisetosa* | COX1 | 617 | PZ852252 |
| DK-0083 | *Lipoptena fortisetosa* | COX1 | 617 | PZ852253 |
| DK-0084 | *Lipoptena fortisetosa* | COX1 | 617 | PZ852254 |
| DK-0085 | *Lipoptena fortisetosa* | COX1 | 617 | PZ852255 |
| DK-0086 | *Lipoptena fortisetosa* | COX1 | 617 | PZ852256 |
| DK-0087 | *Lipoptena fortisetosa* | COX1 | 617 | PZ852257 |
| DK-0088 | *Lipoptena fortisetosa* | COX1 | 617 | PZ852258 |
| DK-0089 | *Lipoptena fortisetosa* | COX1 | 617 | PZ852259 |
| DK-0092 | *Lipoptena fortisetosa* | COX1 | 617 | PZ852260 |
| DK-0093 | *Lipoptena fortisetosa* | COX1 | 617 | PZ852261 |
| DK-0101 | *Lipoptena fortisetosa* | COX1 | 617 | PZ852262 |
| DK-0114 | *Lipoptena fortisetosa* | COX1 | 617 | PZ852263 |
| DK-0115 | *Lipoptena fortisetosa* | COX1 | 617 | PZ852264 |
| DK-0118 | *Lipoptena fortisetosa* | COX1 | 617 | PZ852265 |
| DK-0119 | *Lipoptena fortisetosa* | COX1 | 617 | PZ852266 |
| DK-0126 | *Lipoptena fortisetosa* | COX1 | 617 | PZ852267 |
| DK-0130 | *Lipoptena fortisetosa* | COX1 | 617 | PZ852268 |
| DK-0131 | *Lipoptena fortisetosa* | COX1 | 617 | PZ852269 |
| DK-0132 | *Lipoptena fortisetosa* | COX1 | 617 | PZ852270 |
| DK-0134 | *Lipoptena fortisetosa* | COX1 | 617 | PZ852271 |
| DK-0135 | *Lipoptena fortisetosa* | COX1 | 617 | PZ852272 |
| DK-0136 | *Lipoptena fortisetosa* | COX1 | 617 | PZ852273 |
| DK-0137 | *Lipoptena fortisetosa* | COX1 | 617 | PZ852274 |
| DK-0138 | *Lipoptena fortisetosa* | COX1 | 617 | PZ852275 |
| DK-0140 | *Lipoptena fortisetosa* | COX1 | 617 | PZ852276 |
| DK-0146 | *Lipoptena fortisetosa* | COX1 | 617 | PZ852277 |
| DK-0147 | *Lipoptena fortisetosa* | COX1 | 617 | PZ852278 |
| DK-0153 | *Lipoptena fortisetosa* | COX1 | 617 | PZ852279 |
| DK-0155 | *Lipoptena fortisetosa* | COX1 | 617 | PZ852280 |
| DK-0159 | *Lipoptena fortisetosa* | COX1 | 617 | PZ852281 |
| DK-0160 | *Lipoptena fortisetosa* | COX1 | 617 | PZ852282 |
| DK-0161 | *Lipoptena fortisetosa* | COX1 | 617 | PZ852283 |
| DK-0162 | *Lipoptena fortisetosa* | COX1 | 617 | PZ852284 |
| DK-0170 | *Lipoptena fortisetosa* | COX1 | 617 | PZ852285 |
| DK-0171 | *Lipoptena fortisetosa* | COX1 | 617 | PZ852286 |
| DK-0190 | *Lipoptena fortisetosa* | COX1 | 617 | PZ852287 |
| DK-0191 | *Lipoptena fortisetosa* | COX1 | 617 | PZ852288 |
| DK-0192 | *Lipoptena fortisetosa* | COX1 | 617 | PZ852289 |
| DK-0193 | *Lipoptena fortisetosa* | COX1 | 617 | PZ852290 |
| DK-0195 | *Lipoptena fortisetosa* | COX1 | 617 | PZ852291 |
| DK-0196 | *Lipoptena fortisetosa* | COX1 | 617 | PZ852292 |
| DK-0197 | *Lipoptena fortisetosa* | COX1 | 617 | PZ852293 |
| DK-0198 | *Lipoptena fortisetosa* | COX1 | 617 | PZ852294 |
| DK-0199 | *Lipoptena fortisetosa* | COX1 | 617 | PZ852295 |
| DK-0201 | *Lipoptena fortisetosa* | COX1 | 617 | PZ852296 |
| DK-0202 | *Lipoptena fortisetosa* | COX1 | 617 | PZ852297 |
| DK-0203 | *Lipoptena fortisetosa* | COX1 | 617 | PZ852298 |
| DK-0204 | *Lipoptena fortisetosa* | COX1 | 617 | PZ852299 |
| DK-0205 | *Lipoptena fortisetosa* | COX1 | 617 | PZ852300 |
| DK-0206 | *Lipoptena fortisetosa* | COX1 | 617 | PZ852301 |
| DK-0207 | *Lipoptena fortisetosa* | COX1 | 617 | PZ852302 |
| DK-0210 | *Lipoptena fortisetosa* | COX1 | 617 | PZ852303 |
| DK-0212 | *Lipoptena fortisetosa* | COX1 | 617 | PZ852304 |
| DK-0213 | *Lipoptena fortisetosa* | COX1 | 617 | PZ852305 |
| DK-0215 | *Lipoptena fortisetosa* | COX1 | 617 | PZ852306 |
| DK-0216 | *Lipoptena fortisetosa* | COX1 | 617 | PZ852307 |
| DK-0217 | *Lipoptena fortisetosa* | COX1 | 617 | PZ852308 |
| DK-0218 | *Lipoptena fortisetosa* | COX1 | 617 | PZ852309 |
| DK-0224 | *Lipoptena fortisetosa* | COX1 | 617 | PZ852310 |
| DK-0225 | *Lipoptena fortisetosa* | COX1 | 617 | PZ852311 |
| DK-0226 | *Lipoptena fortisetosa* | COX1 | 617 | PZ852312 |
| DK-0228 | *Lipoptena fortisetosa* | COX1 | 617 | PZ852313 |
| DK-0230 | *Lipoptena fortisetosa* | COX1 | 617 | PZ852314 |
| DK-0231 | *Lipoptena fortisetosa* | COX1 | 617 | PZ852315 |
| DK-0233 | *Lipoptena fortisetosa* | COX1 | 617 | PZ852316 |
| DK-0242 | *Lipoptena fortisetosa* | COX1 | 617 | PZ852317 |
| DK-0243 | *Lipoptena fortisetosa* | COX1 | 617 | PZ852318 |
| DK-0247 | *Lipoptena fortisetosa* | COX1 | 617 | PZ852319 |
| DK-0248 | *Lipoptena fortisetosa* | COX1 | 617 | PZ852320 |
| DK-0249 | *Lipoptena fortisetosa* | COX1 | 617 | PZ852321 |
| DK-0254 | *Lipoptena fortisetosa* | COX1 | 617 | PZ852322 |
| DK-0258 | *Lipoptena fortisetosa* | COX1 | 617 | PZ852323 |
| DK-0259 | *Lipoptena fortisetosa* | COX1 | 617 | PZ852324 |
| DK-0265 | *Lipoptena fortisetosa* | COX1 | 617 | PZ852325 |
| DK-0272 | *Lipoptena fortisetosa* | COX1 | 617 | PZ852326 |
| DK-0273 | *Lipoptena fortisetosa* | COX1 | 617 | PZ852327 |
| DK-0274 | *Lipoptena fortisetosa* | COX1 | 617 | PZ852328 |
| DK-0275 | *Lipoptena fortisetosa* | COX1 | 617 | PZ852329 |
| DK-0276 | *Lipoptena fortisetosa* | COX1 | 617 | PZ852330 |
| DK-0277 | *Lipoptena fortisetosa* | COX1 | 617 | PZ852331 |
| DK-0282 | *Lipoptena fortisetosa* | COX1 | 617 | PZ852332 |
| DK-0296 | *Lipoptena fortisetosa* | COX1 | 617 | PZ852333 |
| DK-0297 | *Lipoptena fortisetosa* | COX1 | 617 | PZ852334 |
| DK-0307 | *Lipoptena fortisetosa* | COX1 | 617 | PZ852335 |
| DK-0308 | *Lipoptena fortisetosa* | COX1 | 617 | PZ852336 |
| DK-0310 | *Lipoptena fortisetosa* | COX1 | 617 | PZ852337 |
| DK-0311 | *Lipoptena fortisetosa* | COX1 | 617 | PZ852338 |
| DK-0317 | *Lipoptena fortisetosa* | COX1 | 617 | PZ852339 |
| DK-0325 | *Lipoptena fortisetosa* | COX1 | 617 | PZ852340 |
| DK-0330 | *Lipoptena fortisetosa* | COX1 | 617 | PZ852341 |
| DK-0331 | *Lipoptena fortisetosa* | COX1 | 617 | PZ852342 |
| DK-0340 | *Lipoptena fortisetosa* | COX1 | 617 | PZ852343 |
| DK-0341 | *Lipoptena fortisetosa* | COX1 | 617 | PZ852344 |
| DK-0352 | *Lipoptena fortisetosa* | COX1 | 617 | PZ852345 |
| DK-0353 | *Lipoptena fortisetosa* | COX1 | 617 | PZ852346 |
| DK-0357 | *Lipoptena fortisetosa* | COX1 | 617 | PZ852347 |
| DK-0358 | *Lipoptena fortisetosa* | COX1 | 617 | PZ852348 |
| DK-0369 | *Lipoptena fortisetosa* | COX1 | 617 | PZ852349 |
| DK-0370 | *Lipoptena fortisetosa* | COX1 | 617 | PZ852350 |
| DK-0373 | *Lipoptena fortisetosa* | COX1 | 617 | PZ852351 |
| DK-0378 | *Lipoptena fortisetosa* | COX1 | 617 | PZ852352 |
| DK-0379 | *Lipoptena fortisetosa* | COX1 | 617 | PZ852353 |
| DK-0380 | *Lipoptena fortisetosa* | COX1 | 617 | PZ852354 |
| DK-0387 | *Lipoptena fortisetosa* | COX1 | 617 | PZ852355 |
| DK-0388 | *Lipoptena fortisetosa* | COX1 | 617 | PZ852356 |
| DK-0389 | *Lipoptena fortisetosa* | COX1 | 617 | PZ852357 |
| DK-0390 | *Lipoptena fortisetosa* | COX1 | 617 | PZ852358 |
| DK-0391 | *Lipoptena fortisetosa* | COX1 | 617 | PZ852359 |
| DK-0392 | *Lipoptena fortisetosa* | COX1 | 617 | PZ852360 |
| DK-0393 | *Lipoptena fortisetosa* | COX1 | 617 | PZ852361 |
| DK-0395 | *Lipoptena fortisetosa* | COX1 | 617 | PZ852362 |
| DK-0396 | *Lipoptena fortisetosa* | COX1 | 617 | PZ852363 |
| DK-0397 | *Lipoptena fortisetosa* | COX1 | 617 | PZ852364 |
| DK-0398 | *Lipoptena fortisetosa* | COX1 | 617 | PZ852365 |
| DK-0399 | *Lipoptena fortisetosa* | COX1 | 617 | PZ852366 |
| DK-0400 | *Lipoptena fortisetosa* | COX1 | 617 | PZ852367 |
| DK-0405 | *Lipoptena fortisetosa* | COX1 | 617 | PZ852368 |
| DK-0413 | *Lipoptena fortisetosa* | COX1 | 617 | PZ852369 |
| DK-0005 | *Theileria* spp. | 18S rRNA | 293 | PZ852376 |
| DK-0013 | *Theileria* spp. | 18S rRNA | 293 | PZ852377 |
| DK-0014 | *Theileria* spp. | 18S rRNA | 293 | PZ852378 |
| DK-0018 | *Theileria* spp. | 18S rRNA | 293 | PZ852379 |
| DK-0021 | *Theileria* spp. | 18S rRNA | 293 | PZ852380 |
| DK-0029 | *Theileria* spp. | 18S rRNA | 293 | PZ852381 |
| DK-0032 | *Theileria* spp. | 18S rRNA | 293 | PZ852382 |
| DK-0036 | *Theileria* spp. | 18S rRNA | 293 | PZ852383 |
| DK-0095 | *Theileria* spp. | 18S rRNA | 293 | PZ852384 |
| DK-0096 | *Theileria* spp. | 18S rRNA | 293 | PZ852385 |
| DK-0121 | *Theileria* spp. | 18S rRNA | 293 | PZ852386 |
| DK-0122 | *Theileria* spp. | 18S rRNA | 293 | PZ852387 |
| DK-0124 | *Theileria* spp. | 18S rRNA | 293 | PZ852388 |
| DK-0125 | *Theileria* spp. | 18S rRNA | 293 | PZ852389 |
| DK-0131 | *Theileria* spp. | 18S rRNA | 293 | PZ852390 |
| DK-0138 | *Theileria* spp. | 18S rRNA | 293 | PZ852391 |
| DK-0140 | *Theileria* spp. | 18S rRNA | 293 | PZ852392 |
| DK-0144 | *Theileria* spp. | 18S rRNA | 293 | PZ852393 |
| DK-0155 | *Theileria* spp. | 18S rRNA | 293 | PZ852394 |
| DK-0158 | *Theileria* spp. | 18S rRNA | 293 | PZ852395 |
| DK-0188 | *Theileria* spp. | 18S rRNA | 293 | PZ852396 |
| DK-0189 | *Theileria* spp. | 18S rRNA | 293 | PZ852397 |
| DK-0196 | *Theileria* spp. | 18S rRNA | 293 | PZ852398 |
| DK-0199 | *Theileria* spp. | 18S rRNA | 293 | PZ852399 |
| DK-0222 | *Theileria* spp. | 18S rRNA | 293 | PZ852400 |
| DK-0243 | *Theileria* spp. | 18S rRNA | 293 | PZ852401 |
| DK-0244 | *Theileria* spp. | 18S rRNA | 293 | PZ852402 |
| DK-0306 | *Theileria* spp. | 18S rRNA | 293 | PZ852403 |
| DK-0309 | *Theileria* spp. | 18S rRNA | 293 | PZ852404 |
| DK-0316 | *Theileria* spp. | 18S rRNA | 293 | PZ852405 |
| DK-0326 | *Theileria* spp. | 18S rRNA | 293 | PZ852406 |
| DK-0328 | *Theileria* spp. | 18S rRNA | 293 | PZ852407 |
| DK-0329 | *Theileria* spp. | 18S rRNA | 293 | PZ852408 |
| DK-0387 | *Theileria* spp. | 18S rRNA | 293 | PZ852409 |
| DK-0398 | *Theileria* spp. | 18S rRNA | 293 | PZ852410 |
| DK-0039 | *Anaplasma capra* | 16S rRNA | 1029 | PZ854640 |
| DK-0008 | *Anaplasma capra* | 16S rRNA | 1029 | PZ854641 |
| DK-0135 | *Coxiella*-like organism | 16S rRNA | 680 | PZ862439 |
| DK-0165 | *Coxiella*-like organism | 16S rRNA | 680 | PZ889905 |
| DK-0248 | *Coxiella*-like organism | 16S rRNA | 680 | PZ889906 |
| DK-0304 | *Coxiella*-like organism | 16S rRNA | 680 | PZ889904 |

**B. Bartonella gltA sequences**

| Specimen ID | Sequence / organism | Target gene | Sequence length (bp) | GenBank accession no. |
| --- | --- | --- | --- | --- |
| DK-0001 | *Bartonella* spp. | gltA | 748 | PZ987084 |
| DK-0002 | *Bartonella* spp. | gltA | 748 | PZ987083 |
| DK-0003 | *Bartonella* spp. | gltA | 748 | PZ987082 |
| DK-0007 | *Bartonella* spp. | gltA | 748 | PZ986991 |
| DK-0010 | *Bartonella* spp. | gltA | 748 | PZ987081 |
| DK-0011 | *Bartonella* spp. | gltA | 748 | PZ987080 |
| DK-0013 | *Bartonella* spp. | gltA | 748 | PZ987079 |
| DK-0014 | *Bartonella* spp. | gltA | 748 | PZ987078 |
| DK-0015 | *Bartonella* spp. | gltA | 748 | PZ987077 |
| DK-0016 | *Bartonella* spp. | gltA | 748 | PZ987076 |
| DK-0019 | *Bartonella* spp. | gltA | 748 | PZ987075 |
| DK-0021 | *Bartonella* spp. | gltA | 748 | PZ987074 |
| DK-0030 | *Bartonella* spp. | gltA | 748 | PZ987073 |
| DK-0039 | *Bartonella* spp. | gltA | 748 | PZ987072 |
| DK-0040 | *Bartonella* spp. | gltA | 748 | PZ987071 |
| DK-0050 | *Bartonella* spp. | gltA | 748 | PZ987070 |
| DK-0051 | *Bartonella* spp. | gltA | 748 | PZ987069 |
| DK-0054 | *Bartonella* spp. | gltA | 748 | PZ986992 |
| DK-0057 | *Bartonella* spp. | gltA | 748 | PZ986993 |
| DK-0063 | *Bartonella* spp. | gltA | 748 | PZ986994 |
| DK-0088 | *Bartonella* spp. | gltA | 748 | PZ986995 |
| DK-0096 | *Bartonella* spp. | gltA | 748 | PZ986996 |
| DK-0099 | *Bartonella* spp. | gltA | 748 | PZ987008 |
| DK-0135 | *Bartonella* spp. | gltA | 748 | PZ986997 |
| DK-0146 | *Bartonella* spp. | gltA | 748 | PZ986998 |
| DK-0163 | *Bartonella* spp. | gltA | 748 | PZ986999 |
| DK-0164 | *Bartonella* spp. | gltA | 748 | PZ987000 |
| DK-0165 | *Bartonella* spp. | gltA | 748 | PZ987001 |
| DK-0167 | *Bartonella* spp. | gltA | 748 | PZ987002 |
| DK-0168 | *Bartonella* spp. | gltA | 748 | PZ987003 |
| DK-0169 | *Bartonella* spp. | gltA | 748 | PZ987004 |
| DK-0170 | *Bartonella* spp. | gltA | 748 | PZ987005 |
| DK-0171 | *Bartonella* spp. | gltA | 748 | PZ987006 |
| DK-0172 | *Bartonella* spp. | gltA | 748 | PZ987007 |
| DK-0173 | *Bartonella* spp. | gltA | 748 | PZ987009 |
| DK-0174 | *Bartonella* spp. | gltA | 748 | PZ987010 |
| DK-0175 | *Bartonella* spp. | gltA | 748 | PZ987011 |
| DK-0176 | *Bartonella* spp. | gltA | 748 | PZ987012 |
| DK-0178 | *Bartonella* spp. | gltA | 748 | PZ987013 |
| DK-0179 | *Bartonella* spp. | gltA | 748 | PZ987014 |
| DK-0180 | *Bartonella* spp. | gltA | 748 | PZ987015 |
| DK-0181 | *Bartonella* spp. | gltA | 748 | PZ987016 |
| DK-0182 | *Bartonella* spp. | gltA | 748 | PZ987017 |
| DK-0183 | *Bartonella* spp. | gltA | 748 | PZ987018 |
| DK-0184 | *Bartonella* spp. | gltA | 748 | PZ987019 |
| DK-0185 | *Bartonella* spp. | gltA | 748 | PZ987020 |
| DK-0186 | *Bartonella* spp. | gltA | 748 | PZ987021 |
| DK-0187 | *Bartonella* spp. | gltA | 748 | PZ987022 |
| DK-0188 | *Bartonella* spp. | gltA | 748 | PZ987023 |
| DK-0189 | *Bartonella* spp. | gltA | 748 | PZ987024 |
| DK-0192 | *Bartonella* spp. | gltA | 748 | PZ987025 |
| DK-0193 | *Bartonella* spp. | gltA | 748 | PZ987026 |
| DK-0194 | *Bartonella* spp. | gltA | 748 | PZ987027 |
| DK-0195 | *Bartonella* spp. | gltA | 748 | PZ987028 |
| DK-0198 | *Bartonella* spp. | gltA | 748 | PZ987029 |
| DK-0199 | *Bartonella* spp. | gltA | 748 | PZ987030 |
| DK-0226 | *Bartonella* spp. | gltA | 748 | PZ987031 |
| DK-0227 | *Bartonella* spp. | gltA | 748 | PZ987032 |
| DK-0228 | *Bartonella* spp. | gltA | 748 | PZ987033 |
| DK-0231 | *Bartonella* spp. | gltA | 748 | PZ987034 |
| DK-0233 | *Bartonella* spp. | gltA | 748 | PZ987035 |
| DK-0276 | *Bartonella* spp. | gltA | 748 | PZ987036 |
| DK-0277 | *Bartonella* spp. | gltA | 748 | PZ987037 |
| DK-0278 | *Bartonella* spp. | gltA | 748 | PZ987038 |
| DK-0279 | *Bartonella* spp. | gltA | 748 | PZ987039 |
| DK-0280 | *Bartonella* spp. | gltA | 748 | PZ987040 |
| DK-0281 | *Bartonella* spp. | gltA | 748 | PZ987041 |
| DK-0288 | *Bartonella* spp. | gltA | 748 | PZ987042 |
| DK-0293 | *Bartonella* spp. | gltA | 748 | PZ987043 |
| DK-0295 | *Bartonella* spp. | gltA | 748 | PZ987044 |
| DK-0298 | *Bartonella* spp. | gltA | 748 | PZ987045 |
| DK-0300 | *Bartonella* spp. | gltA | 748 | PZ987046 |
| DK-0301 | *Bartonella* spp. | gltA | 748 | PZ987047 |
| DK-0303 | *Bartonella* spp. | gltA | 748 | PZ987048 |
| DK-0305 | *Bartonella* spp. | gltA | 748 | PZ987049 |
| DK-0306 | *Bartonella* spp. | gltA | 748 | PZ987050 |
| DK-0312 | *Bartonella* spp. | gltA | 748 | PZ987051 |
| DK-0313 | *Bartonella* spp. | gltA | 748 | PZ987052 |
| DK-0314 | *Bartonella* spp. | gltA | 748 | PZ987053 |
| DK-0315 | *Bartonella* spp. | gltA | 748 | PZ987054 |
| DK-0316 | *Bartonella* spp. | gltA | 748 | PZ987055 |
| DK-0317 | *Bartonella* spp. | gltA | 748 | PZ987056 |
| DK-0318 | *Bartonella* spp. | gltA | 748 | PZ987057 |
| DK-0324 | *Bartonella* spp. | gltA | 748 | PZ987058 |
| DK-0341 | *Bartonella* spp. | gltA | 748 | PZ987059 |
| DK-0342 | *Bartonella* spp. | gltA | 748 | PZ987060 |
| DK-0343 | *Bartonella* spp. | gltA | 748 | PZ987061 |
| DK-0344 | *Bartonella* spp. | gltA | 748 | PZ987062 |
| DK-0360 | *Bartonella* spp. | gltA | 748 | PZ987063 |
| DK-0370 | *Bartonella* spp. | gltA | 748 | PZ987064 |
| DK-0371 | *Bartonella* spp. | gltA | 748 | PZ987065 |
| DK-0372 | *Bartonella* spp. | gltA | 748 | PZ987066 |
| DK-0390 | *Bartonella* spp. | gltA | 748 | PZ987067 |
| DK-0406 | *Bartonella* spp. | gltA | 748 | PZ987068 |

The COX1 population-genetic dataset comprised 617 bp; the COX1 phylogenetic tree was based on a 577-bp common alignment after inclusion and trimming of reference sequences. Two chromatograms showing mixed A/G peaks were excluded from genotype assignment and phylogenetic analysis.
